# Real‐Time Dissection of the Transportation and miRNA‐Release Dynamics of Small Extracellular Vesicles

**DOI:** 10.1002/advs.202205566

**Published:** 2023-01-04

**Authors:** Hou‐Fu Xia, Zi‐Li Yu, Li‐Juan Zhang, Shu‐Lin Liu, Yi Zhao, Jue Huang, Dan‐Dan Fu, Qi‐Hui Xie, Hai‐Ming Liu, Zhi‐Ling Zhang, Yi‐Fang Zhao, Min Wu, Wei Zhang, Dai‐Wen Pang, Gang Chen

**Affiliations:** ^1^ The State Key Laboratory Breeding Base of Basic Science of Stomatology (Hubei‐MOST) and Key Laboratory of Oral Biomedicine Ministry of Education School and Hospital of Stomatology Wuhan University Wuhan 430079 P. R. China; ^2^ Department of Oral and Maxillofacial Surgery School and Hospital of Stomatology Wuhan University Wuhan 430079 P. R. China; ^3^ College of Chemistry and Molecular Sciences Wuhan University Wuhan 430072 P. R. China; ^4^ State Key Laboratory of Medicinal Chemical Biology Tianjin Key Laboratory of Biosensing and Molecular Recognition Research Center for Analytical Sciences and College of Chemistry Nankai University Tianjin 300071 P. R. China; ^5^ Department of Prosthodontics School and Hospital of Stomatology Wuhan University Wuhan 430079 P. R. China; ^6^ TaiKang Center for Life and Medical Sciences Wuhan University Wuhan 430071 P. R. China; ^7^ Frontier Science Center for Immunology and Metabolism Wuhan University Wuhan 430071 P. R. China

**Keywords:** angiogenesis, extracellular vesicles, miRNA, quantum dots, single particle tracking

## Abstract

Extracellular vesicles (EVs) are cell‐derived membrane‐enclosed structures that deliver biomolecules for intercellular communication. Developing visualization methods to elucidate the spatiotemporal dynamics of EVs’ behaviors will facilitate their understanding and translation. With a quantum dot (QD) labeling strategy, a single particle tracking (SPT) platform is proposed here for dissecting the dynamic behaviors of EVs. The interplays between tumor cell‐derived small EVs (T‐sEVs) and endothelial cells (ECs) are specifically investigated based on this platform. It is revealed that, following a clathrin‐mediated endocytosis by ECs, T‐sEVs are transported to the perinuclear region in a typical three‐stage pattern. Importantly, T‐sEVs frequently interact with and finally enter lysosomes, followed by quick release of their carried miRNAs. This study, for the first time, reports the entire process and detailed dynamics of T‐sEV transportation and cargo‐release in ECs, leading to better understanding of their proangiogenic functions. Additionally, the QD‐based SPT technique will help uncover more secrets of sEV‐mediated cell–cell communication.

## Graphical abstract

By developing the quantum dot‐based multicomponent labeling strategy and assembling the single particle tracking platform, for the first time, the three‐stage transportation process is tracked following the clathrin‐mediated endocytosis of tumor‐derived small extracellular vesicles (sEVs) in endothelial cells and their miRNA cargo‐release details are recorded further in real time.

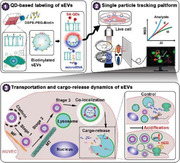

## 1 Introduction

Extracellular vesicles (EVs) are lipid bilayer‐enclosed structures secreted by a variety of cells and are enriched with plenty of biomolecules (e.g., proteins, miRNAs). EVs comprise heterogeneous populations that vary in sizes and origins, including small EVs (sEVs, < 200 nm) (e.g., exosomes, which formed inside endosomal compartments) and large EVs (lEVs, > 200 nm) (e.g., microvesicles, which formed by direct budding from the plasma membrane).^[^ ^1^ ^]^ Over the past two decades, EVs have emerged as important mediators of intercellular communication due to their inherent capacity to carry and deliver various bioactive molecules between cells.^[^ ^2^ ^]^ EVs have also been gradually recognized as critical players in a wide range of diseases including malignant tumors.^[^ ^3^, ^4^ ^]^ In the meantime, since they can cross multiple biological barriers such as the blood–brain barrier and be taken up by their target cells with a high efficiency and specificity, EVs are recently of immense interest as biological vectors for therapeutic drug delivery.^[^ ^5^, ^6^ ^]^ Considering the important functions of EVs in both physiological and pathological settings as well as their great potential for drug delivery, there is a growing need to characterize the cellular and molecular underpinnings of EV transportation and cargo delivery into their recipient cells. Thus, significant research efforts have recently been dedicated to developing methods for EV imaging, especially the direct optical visualization and tracking of EVs.^[^ ^7^, ^8^ ^]^ Although the development of high‐resolution microscopes have already provided powerful tools for the live imaging of EVs,^[^ ^9^, ^10^ ^]^ subcellular tracking of EVs, especially sEVs, with high specificity and accuracy can be still challenging owing to the major drawbacks in current labeling methods.

Different from microsized cells, most EVs are nanosized particles that often dynamically interact with their recipient cells at the subcellular levels.^[^ ^11^ ^]^ An ideal real‐time and long‐term optical tracking of EVs’ behaviors in their recipient cells often demands high‐quality fluorescent labeling of EVs to ensure the accuracy and reproducibility.^[^ ^8^ ^]^ First, the labeling must provide strong and stable signals to support fast imaging and long‐term tracking of the nanosized EVs. Second, the labeling should cover the whole interested population of EVs, and meanwhile be convenient and biofriendly enough for the maximum preservation of their natural properties. Third, the labeling should be highly specific to the EVs for discriminating them from the intrinsic membrane structures of recipient cells. Last but not least, the labeling material as well as the produced signals should have a synchronous lifespan with that of EVs to avoid false positive signals. However, the most currently employed two labeling methods for EVs, i.e., the fluorescent protein‐ and organic dye‐based labeling, were initially designed and used for cells. They are still far from the above criteria to support an ideal subcellular tracking of EVs.^[^ ^8^, ^12^ ^]^ For the labeling by conjugating fluorescent proteins to the marker proteins of EVs (e.g., CD63, CD81, etc.), the signal intensity is highly dependent on the expression level of the fluorescent proteins, thus bringing uncertainties to the quality of subsequent imaging.^[^ ^13^, ^14^ ^]^ Also, the genetic modification process and excessive expression of fluorescent proteins are under suspicion for affecting the cargo contents and biological behaviors of EVs. Importantly, the fluorescent protein‐based labeling may be limited to only a few subpopulations of EVs that express the respective markers, thereby impeding the general population study of EVs. On the other hand, a wide range of organic fluorescent dyes such as PKH26, DiR andcarboxyfluorescein diacetate succinimidyl ester (CFSE), which can be nonspecifically incorporated into the lipid bilayers or protein components present in EVs, are universal to label EVs of different subtypes.^[^ ^15^, ^16^ ^]^ However, the application of these fluorescent dyes especially in the field of real‐time dynamic tracking is often restricted by their drawbacks such as weak intensity and quick photobleaching. In addition, most of the fluorescent dyes have very long half‐times and may produce false signals due to aggregation or micelle formation, thereby causing misunderstandings of EVs’ behaviors.

Quantum dots (QDs) are inorganic semiconductor nanomaterials that possess a plethora of advantages over fluorescent proteins and organic dyes, including strong fluorescent intensity and negligible photobleaching, narrow emission and broad excitation spectra, tunable emission peaks, and the ability to be functionalized by different biotags.^[^ ^17^ ^]^ These superior properties of QDs have placed them in an advantageous position in the field of high‐resolution live imaging, especially for single particle tracking (SPT) of nanosized organisms.^[^ ^18^ ^]^ Compared with traditional cellular imaging strategies, single particle tracking is a powerful technique to study the dynamic and complex biological processes in live cells in real time, permitting characterization of the unique behaviors of each heterogeneous individual.^[^ ^19^ ^]^ The strong and stable signals provided by QDs are essential for the sensitive detection, quick imaging, and long‐term investigation during single particle tracking. Also, the large stokes shifts and broad absorption spectra of QDs allow simultaneous excitation of multiple probes whose emission wavelengths can be continuously tuned by modifying particle size or chemical composition. Meanwhile, a diversity of surface functionalization makes it easy for efficient QD‐based labeling of most types of targets.^[^ ^18^, ^20^ ^]^ Of note, in an effort to reveal the in vivo behaviors of EVs, we have conducted several pioneering studies to realize the reliable and biofriendly labeling of EVs with QDs.^[^ ^21^, ^22^ ^]^ By either a donor cell‐assisted membrane biotinylation strategy or an electroporation‐mediated loading strategy, we have successfully achieved the efficient QD labeling of multiple EV subtypes with prominent advantages, including convenient procedure, high specificity, and good biocompatibility. Relying on the valuable traceability endowed by QD labeling, we have assessed the EVs’ dynamic biodistribution and protumoral behaviors in vivo by locating the EVs in live animals with high‐resolution.^[^ ^21^, ^22^, ^23^ ^]^ However, neither our previous nor others’ studies tracked the EVs’ dynamic behaviors in the long‐term interaction, not to mention how EVs were taken by the recipient cells and how the cargos were released.^[^ ^21^, ^22^, ^23^, ^24^ ^]^ Therefore, a QD‐based single particle tracking platform for studying the spatiotemporal dynamics of EVs’ behaviors in recipient cells is still to be developed.

In the present study, therefore, we aimed to take one step forward to establish a universal QD‐based single particle tracking platform to dissect the dynamic behaviors of EVs in real time and long term at the subcellular level. Based on a further optimization of our previously reported strategy, the membranes of EVs were specifically labeled with QDs. By taking advantage of the superior properties of the QD‐labeled EVs, we assembled a single particle tracking platform capable of high‐speed imaging, long‐term monitoring and automated data processing. With this QD‐based single particle tracking platform, we witnessed and dissected the entire transportation and miRNA‐release process of T‐sEVs in their recipient endothelial cells (ECs). Dynamic tracking and detailed analysis of the motion trajectories of multiple individual T‐sEVs revealed that, following an endocytosis‐mediated entry into ECs, T‐sEVs exhibited a typical cytoskeleton‐dependent “slow‐fast‐slow” three‐stage transportation in the cell, namely, a first slow movement in the cell periphery, then a rapid trafficking to the cell nucleus, and a final long‐time confined motion in the perinuclear region. Simultaneous tracking of multilabeled T‐sEVs and the recipient cell endosomes further revealed that T‐sEVs in the perinuclear region frequently interacted with and finally entered the lysosomes, followed by a quick release of their carried miRNAs. The QD‐based single particle tracking platform developed in this study provides a versatile and powerful tool for studying the cargo‐delivery behaviors of EVs, which would help to better understand EV‐mediated cell–cell communication.

## 2 Results and Discussion

### 2.1 Membrane Biotinylation of Multiple EV Subtypes

By culturing the donor cells in medium containing biotin‐functionalized 1,2‐distearoyl‐sn‐glycero‐3‐phosphoethanolamine‐*N*‐(polyethylene glycol) (DSPE‐PEG‐Biotin), an amphiphilic molecule that can self‐assemble into the plasma membrane,^[^ ^25^ ^]^ our previous studies have successfully modified the derived EV membranes with biotin, providing a versatile platform for labeling EVs with multiple streptavidin (SA)‐conjugated nanomaterials including QDs.^[^ ^26^, ^27^, ^28^, ^29^ ^]^ Since nearly all EVs are derived from their donor cells’ plasma membrane either by outward budding or via inward invagination, this membrane biotinylation strategy would be applicable to multiple subtypes of EVs. Here, with a dedicated flow cytometer capable of detecting nanosized particles,^[^ ^30^ ^]^ we could specifically focus on different subtypes of EVs (Figure S1a–c, Supporting Information). Using oral cancer CAL27 cells as the model donors, we revealed that incubation of the cells with DSPE‐PEG‐Biotin led to successful membrane biotinylation of both the derived sEVs and lEVs in a time‐ and concentration‐dependent manner (Figure S2a–c, Supporting Information). More than 95% of sEVs and lEVs were biotinylated after culturing their donor cells in medium containing 30 µg mL^−1^ DSPE‐PEG‐Biotin for 6 d. In addition, the membrane biotinylation efficiency showed no significant difference between the sEVs of different cellular origins (Figure S2d, Supporting Information). Moreover, the results of nanoparticle tracking analysis (NTA) verified that the secretion pattern and production of sEVs by biotinylated donor cells were nearly unchanged compared to the control cells (Figure S3a, Supporting Information). The size distribution and morphology of sEVs from biotinylated donor cells was comparable to that of the control group (Figure S3b,c, Supporting Information). Also, membrane biotinylation did not influence the expression patterns of the characteristic proteins (Figure S3d,f, Supporting Information) or miRNAs (Figure S3g, Supporting Information) in the sEVs. These results clarify that the membrane biotinylation strategy can serve as an efficient, universal and biofriendly approach for modifying multiple subtypes of EVs.

### 2.2 Membrane‐Specific Labeling of sEVs with QDs

**Figure 1:**
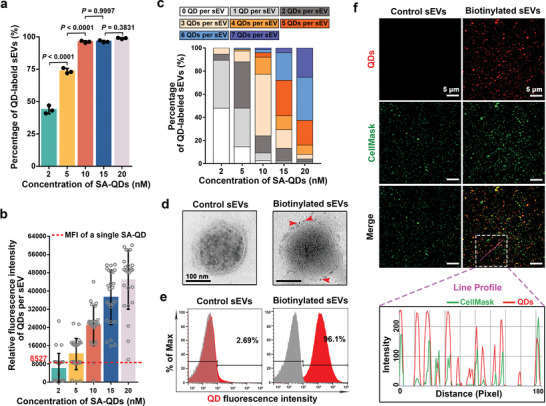
Membrane‐specific labeling of biotinylated sEVs with QDs. a) Flow cytometric analysis of the labeling efficiency of biotinylated sEVs after incubation with SA‐QDs at indicated concentration (*n* = 3), data are presented as mean ± SD, significance was determined using one‐way analysis of variance (ANOVA) followed by Tukey's multiple comparison test. b) Confocal microscopy analysis of the relative fluorescence intensity of multiple single biotinylated sEV after incubation with SA‐QDs at indicated concentration (*n* = 25), data are presented as mean ± SD. The red dashed line represents the mean fluorescence intensity (MFI) of a single SA‐QD under the same condition. c) Quantifying the number of QDs conjugated to the membrane of sEVs after incubation with SA‐QDs at indicated concentration by analyzing the fluorescence intensity of individual sEV particles (*n* = 25). d) TEM images of control sEVs and biotinylated sEVs after incubation with SA‐QDs at 10 nm. e) Flow cytometric analysis of control sEVs and biotinylated sEVs after incubation with SA‐QDs at 10 nm (*n* = 3). f) Fluorescence images of control sEVs and biotinylated sEVs after incubation with SA‐QDs (red) followed by CellMask (green) staining, line profile showed the distribution of SA‐QD (red) and CellMask (green) signals on the purple line.

By taking advantage of the specific interaction and high affinity between biotin and SA, sEVs with biotinylated membranes can be conveniently and directly labeled with SA‐conjugated QDs (SA‐QDs).^[^ ^31^ ^]^ Real‐time high‐resolution single particle tracking of sEVs makes strict demands on the QD‐based labeling strategy. Even though the accumulation of multiple QDs could directly improve imaging quality by increasing signal intensity and signal‐to‐noise ratio,^[^ ^32^ ^]^ too many QDs on the membrane surface of sEVs would potentially cause undesirable effects on their biological behaviors. That means, the ideal labeling strategy of sEVs based on QDs should focus on balancing the contradiction caused by the number of QDs between image quality and external interference. We therefore investigated the optimal dosage of QDs for labeling sEVs, aiming for an ideal labeling with controllable and appropriate number of QDs. For this purpose, a fixed amount of biotinylated sEVs were incubated with different concentrations of SA‐QDs in 100 µL phosphate buffered saline (PBS), followed by removing excess free SA‐QDs via sucrose gradient centrifugation. The results of high‐resolution flow cytometry (FCM) confirmed that the highest labeling efficiency can be achieved at a concentration greater than 10 nm (**Figure** **1**a). Due to the excellent dispersity and uniform fluorescence intensity of each single QD particle, the number of QDs conjugated to the membranes of sEVs was further quantitatively assessed by analyzing the fluorescence intensity of individual sEV particles. It was shown that the fluorescence intensity of each sEV, reflecting the number of QDs on sEV surface, was closely associated with the labeling concentration of QDs (Figure 1b). More than 92% of the biotinylated sEVs carried less than 5 QDs when labeled with SA‐QDs at 10 nm, specifically, about 88% of which carried 2–4 QDs (Figure 1c). The increased concentration of SA‐QDs over 10 nm, which failed to further improve the labeling efficiency, merely aggravated the burden of a single sEV. About 64% of the biotinylated sEVs carried more than 4 QDs after incubation with SA‐QDs at 15 nm. For the optimal labeling and best performance in single particle tracking, sEVs labeled with SA‐QDs at 10 nm were used in the subsequent studies. As confirmed by transmission electron microscopy (TEM) images (Figure 1d), the biotinylated sEVs were surrounded by about 3 QDs after incubation with SA‐QDs at 10 nm, whereas no SA‐QD could be observed on the control sEVs. The results of FCM also verified more than 96% of the biotinylated sEVs were specifically labeled (Figure 1e). Some of the QD‐labeled sEVs were further incubated with CellMask Green, an organic fluorescent dye for plasma membrane staining, to further obtain dual‐labeled sEVs. As shown in Figure 1f, almost all the QD signals were colocalized with the green CellMask signals in the biotinylated sEVs, whereas nearly no QD signals could be observed in the control sEVs, which was consistent with the result of line profile analysis. These above results demonstrate the achievement of membrane‐specific labeling of sEVs with appropriate number of QDs by conveniently tuning the ratio of SA‐QDs to the biotinylated sEVs. In addition, the membrane‐specific labeling of T‐sEVs with QDs was further confirmed to have little influence on T‐sEVs’ intrinsic proangiogenic function (Figure S4, Supporting Information). This reaffirms the great biocompatibility of our QD‐based EV labeling strategy.

### 2.3 Superiority of the QD‐Labeled sEVs for Single Particle Tracking

**Figure 2:**
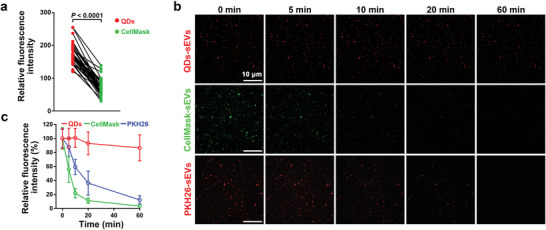
Superiority of the QD‐labeled sEVs for single particle tracking. a) Matched‐pair analysis of the fluorescence intensity of QDs and CellMask from each dual‐labeled sEV particle (*n* = 40), significance was determined using Wilcoxon matched‐pairs signed rank test. b) Representative fluorescence images of QD‐, CellMask‐ or PKH26‐labeled sEVs after continuous irradiation for different time. c) The fluorescence intensity of QD‐, CellMask‐, or PKH26‐labeled sEVs after continuous irradiation for different time (*n* = 15), data are presented as mean ± SD.

As aforementioned, strong and stable fluorescence signals are essential for the real‐time and high‐resolution tracking of sEVs. Thus, we then tested the signal intensity and stability of QD‐labeled sEVs. As shown in **Figure** **2**a, the fluorescence intensity of QD‐labeled sEVs was much higher than that of CellMask‐labeled sEVs. Importantly, after a continuous illumination under the confocal microscope for 60 min, the fluorescence signals of QD‐labeled sEVs were still very intense; however, the signals of CellMask‐ or PKH26‐labeled sEVs became weaker after only 10 min and could only be faintly detected after 20 min (Figure 2b,c). These results suggest that labeling of sEVs with only 2–4 QDs can provide stronger and more stable signals than organic dye to support quick imaging and long‐term tracking. These advantageous properties of QD‐labeled sEVs for single particle tracking would provide an unprecedented opportunity to dissect the subcellular behaviors of sEVs.

### 2.4 The Single Particle Tracking Platform for QD‐Labeled sEVs

**Figure 3:**
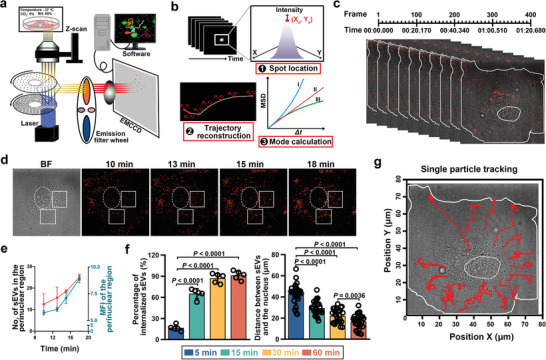
Establishment of the single particle tracking system for QD‐labeled T‐sEVs. a) Schematic of the QD‐based single particle tracking platform. The colored arrows represent the optical path. b) Data analysis process for the subcellular behaviors of individual sEV particles. c) A representative imaging and tracking of QD‐labeled T‐sEV particles in individual EC. d) Snapshots of the transportation of QD‐labeled T‐sEVs in a live EC. The white dotted ellipse represents the nucleus and the white rectangular boxes indicate the perinuclear regions. e) The number and corresponding mean fluorescence intensity (MFI) of QD‐labeled T‐sEVs in the perinuclear region over time (*n* = 4). f) The quantitative analysis about the internalization (*n* = 5) and transportation (*n* = 30) of T‐sEVs at different incubation time, data are presented as mean ± SD, significance was determined using one‐way ANOVA followed by Tukey's multiple comparison test. g) A representative schematic of the reconstructed trajectories of multiple T‐sEVs in one EC.

To better understand the transportation and cargo‐delivery behaviors of sEVs, we assembled a single particle tracking platform for high‐speed imaging and long‐term monitoring of QD‐labeled sEVs at the subcellular level. As shown in **Figure** **3**a, this single particle tracking platform was equipped with a spinning‐disk confocal microscope, an electron multiplying charge coupled device, and an online live cell culture device. Meanwhile, an automated strategy, being capable of spot location, trajectory reconstruction and mode calculation, was utilized to analyze the subcellular behaviors of individual EV particles (Figure 3b). With this QD‐based single particle tracking platform, we attempted to investigate the dynamic behaviors of sEVs in their recipient cells. Given the growing attention on T‐sEVs, especially their interplay with normal stromal cells,^[^ ^33^ ^]^ we here selected human oral cancer cell‐derived sEVs as the model sEVs and human umbilical vein endothelial cells (HUVECs) as their model recipient cells, aiming for a better understanding of the mechanisms underlying tumor‐induced angiogenesis. As shown in Figure 3c, multiple T‐sEV particles labeled with QDs were tracked simultaneously and dynamically in individual cell with a short frame interval (200 ms). It was found that the number of T‐sEVs entered the EC and traveled toward the nucleus were increased over time (Figure 3d,e). Of interest, the quantification analysis based on the results of different coculture time demonstrated that more than 65.33% of T‐sEVs at 15 min and 87.25% of T‐sEVs at 30 min had translocated into the cytoplasm, and the average distance between sEVs and the nucleus was reduced by 60% from 5 to 30 min (Figure 3f), suggesting the rapid and efficient internalization behaviors. For more detailed information, the motion patterns of multiple T‐sEVs within 15 min were then analyzed individually. The reconstructed movement trajectories revealed that T‐sEVs entered into the EC from different directions but rapidly converged to the perinuclear region in a unidirectional manner (Figure 3g). The results demonstrate the successful establishment of a QD‐based single particle tracking platform for EV studies, and more importantly suggest that the transportation of T‐sEVs in ECs was a well‐regulated process showing specific dynamics in both spatial and temporal dimensions, thus warranting a further dissection.

### 2.5 Clathrin‐Mediated Endocytic Entry of T‐sEVs into ECs

**Figure 4:**
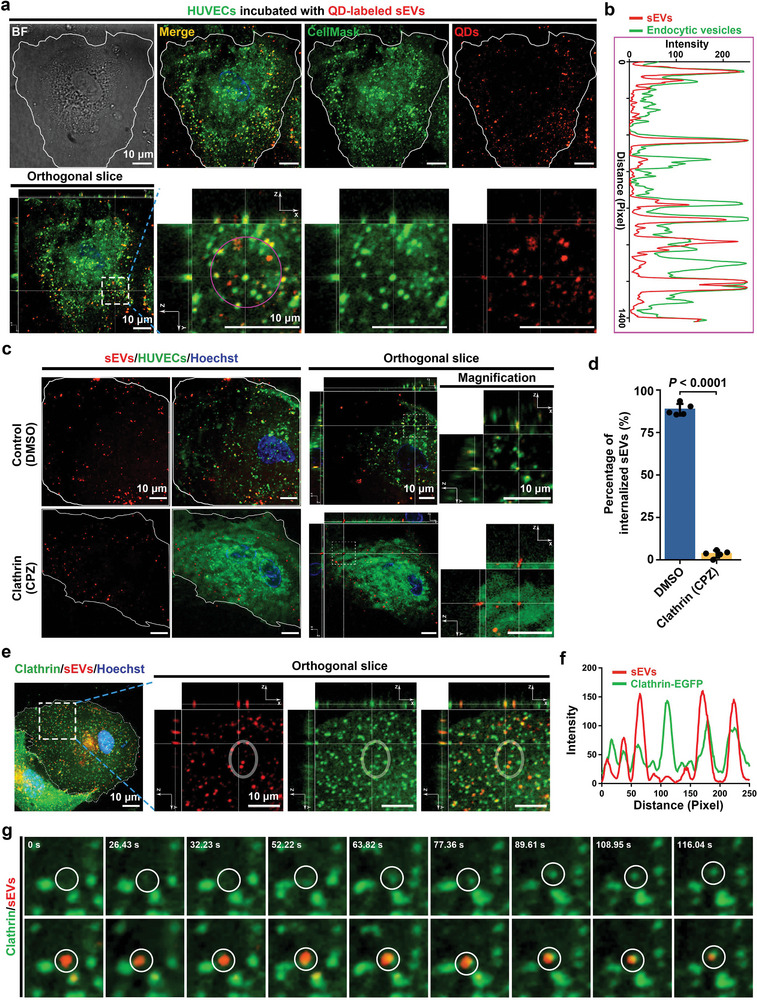
Clathrin‐mediated endocytic entry of T‐sEVs into ECs. a) Representative orthogonal slice images of QD‐labeled T‐sEVs in a CellMask‐labeled EC, nearly all the T‐sEVs were colocalized with endocytic vesicles. b) Line profile analysis indicated the distribution of endocytic vesicle (green) and T‐sEV (red) signals on the purple circle shown in (a). c) Representative confocal fluorescence images showing the effects of inhibiting clathrin‐mediated endocytosis on the internalization of T‐sEVs by ECs. Control, DMSO (Dimethylsulfoxide); Clathrin, CPZ (Chlorpromazine, 100 µm). d) Quantitative analysis of the endocytosis of T‐sEVs by ECs shown in (c) (*n* = 5), data are presented as mean ± SD, significance was determined using two tailed unpaired *t*‐test. e) Representative confocal fluorescence images showing the colocalization of T‐sEVs with EGFP‐labeled clathrin clusters in ECs. f) Line profile analysis indicated the colocalization of clathrin (green) and T‐sEV (red) signals on the white circle shown in (e). g) Snapshots of the process of the clathrin gradually wrapping a T‐sEV around to form a distinct unit as shown in Video S2 in the Supporting Information.

For a comprehensive understanding of the internalization mechanisms of sEVs, we first investigated the entry route of sEVs into their recipient cells. The recipient ECs were stained with CellMask to light their cytomembrane and then cultured with QD‐labeled T‐sEVs. The fluorescence images (**Figure** **4**a) and corresponding line profile analysis (Figure 4b) showed an efficient colocalization between QD and CellMask signals in the cytoplasm, suggesting the internalization of T‐sEVs by plasma membrane‐derived endocytic vesicles. Moreover, the coincident movements of QD‐labeled EVs and CellMask‐positive endocytic vesicles indicated that endocytic pathway was involved in the internalization of T‐sEVs by ECs (Video S1, Supporting Information). To gain more insights into the above findings, specific inhibitors for different endocytic pathways were employed. As shown in Figure 4c,d, inhibition of clathrin‐mediated endocytosis by chlorpromazine (CPZ) nearly abolished the colocalization of T‐sEVs with endocytic vesicles. However, specific blockade of macropinocytosis or caveolae‐mediated endocytosis exhibited little effect (Figure S5, Supporting Information). These results suggested that clathrin‐mediated endocytosis is essential for the internalization of T‐sEVs by ECs. In addition, coculture experiments based on temperature gradient revealed that low temperature significantly inhibited the endocytosis of T‐sEVs by ECs (Figure S6, Supporting Information), confirming the energy‐dependent manner of the T‐sEV internalization by ECs. The endocytosis‐dependent entry of T‐sEVs into ECs was also confirmed by colocalization of T‐sEVs with clathrin^+^ clusters, showing the enhanced green fluorescent protein (EGFP)‐labeled clathrin gradually proceeded toward the absorbed sEV on the cellular membrane and eventually wrapped it around to form a distinct unit (Figure 4e–g and Video S2, Supporting Information).

### 2.6 Three‐Stage Transportation of T‐sEVs in ECs

**Figure 5:**
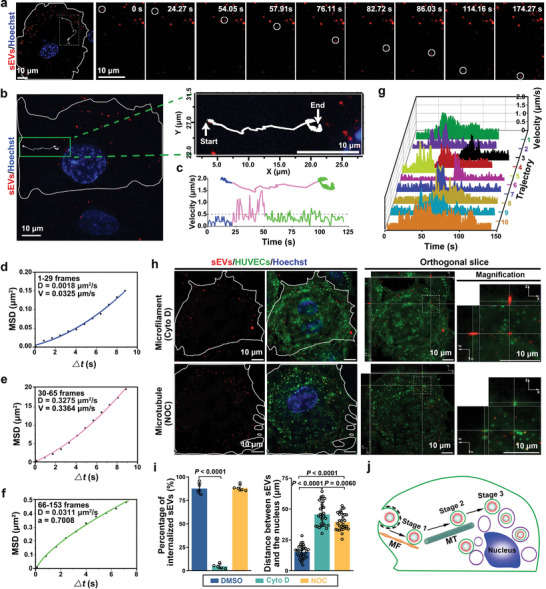
A typical three‐stage transportation pattern of T‐sEVs in ECs. a) Snapshots of the transportation process of a T‐sEV (red) in EC as shown in Video S3 in the Supporting Information. b) The movement trajectory of the T‐sEV (red) shown in Video S4 in the Supporting Information. c) The movement trajectory and instantaneous speed of the T‐sEV shown in Video S5 in the Supporting Information. d–f) MSD‐time interval plots of the T‐sEV in d) stage 1, e) stage 2, and f) stage 3 of the transportation process. g) The instantaneous speed‐time plots of the transportation process of ten representative T‐sEVs in ECs. h) Representative confocal fluorescence images of T‐sEV (red) transportation in ECs (green) treated with microfilament inhibitor and microtubule inhibitor. Microfilament inhibitor, Cyto D (Cytochalasin D, 2 µm); Microtubule inhibitor, NOC (Nocodazole, 25 µm). i) Quantitative analysis of the internalization (*n* = 5) and transportation (*n* = 30) of T‐sEVs in ECs shown in (h), data are presented as mean ± SD, significance was determined using one‐way ANOVA followed by Tukey's multiple comparison test. j) Schematic illustration of the cytoskeleton‐mediated three‐stage transportation pattern of T‐sEVs in ECs. MF (Microfilament), MT (microtubule), the purple circles represent other structures in the perinuclear region.

To dissect the intracellular transportation dynamics of T‐sEVs after their entry into the recipient cells, we analyzed the motion trajectories of multiple T‐sEVs in individual ECs in detail. **Figure** **5**a,b displays the typical transportation process of a single T‐sEV, where the T‐sEV moved directionally in the cytoplasm after their entry into the EC and finally reached the perinuclear region (Videos S3 and S4, Supporting Information). The time trajectories of the position and velocity of the T‐sEV are shown in Figure 5c. These results illustrated a previously unknown three‐stage transportation process showing an obvious “slow‐fast‐slow” pattern (Video S5, Supporting Information). Stage 1 was a slow and time‐consuming movement in the cell periphery region. As shown in the upward‐curving mean square displacement (MSD)‐time interval plot (Figure 5d), the diffusion coefficient (*D*) and fitting velocity (*V*) were 0.0018 µm^2^ s^−1^ and 0.0325 µm s^−1^, respectively, suggesting that it was a typical slow directed motion. Stage 2 was a fast unidirectional movement from the cell periphery to the cell nucleus following stage 1. The calculation of MSD on time interval demonstrated it as a typical fast directed motion with the *D* and *V* value of 0.3275 µm^2^ s^−1^ and 0.3364 µm s^−1^, respectively (Figure 5e). Stage 3 was a slow confined movement in the perinuclear region. The MSD‐time interval plot suggested it was an anomalous diffusion, and the *D* and *α* value was 0.0311 µm^2^ s^−1^ and 0.7008, respectively (Figure 5f). Notably, by analyzing the individual time trajectories of multiple T‐sEVs trafficking in ECs, we further determined that this “slow‐fast‐slow” three‐stage transportation was a coordinated population behavior shared by most of the internalized T‐sEVs (Figure 5g). For further understanding of this distinct three‐stage motion pattern of T‐sEVs, we then investigated the mechanisms involved in the T‐sEV movement. Considering the predominant role of cytoskeleton in membrane trafficking of various components in live cells, the microfilament inhibitor cytochalasin D and the microtubule inhibitor nocodazole (NOC) were employed here. As shown in Figure 5h,i, cytochalasin D significantly disturbed the movement of T‐sEVs at stage I, as characterized by the decreased internalization of T‐sEVs and their limited mobility in the cell periphery region. Nocodazole did not affect the colocalization efficiency between T‐sEVs and endocytic vesicles in ECs, while significantly prevented the converge of T‐sEVs to the cell nucleus, leading to an accumulation of internalized T‐sEVs in the cytoplasm. Those results indicated that microfilaments were mainly involved in endocytosis and the following slow transportation of T‐sEVs at the edge of ECs in stage 1, while the fast and long‐time transport process (Stage 2) in the cytoplasm were mainly dependent on microtubules (Figure 5j). Previous studies have revealed that cargos travel along microfilaments normally at a speed less than 0.5 µm s^−1^, while move faster along microtubules at a speed of several µm s^−1^.^[^ ^34^, ^35^ ^]^ Our data suggest that the instantaneous velocity of T‐sEVs movement was persistently lower than 0.5 µm s^−1^ at stage 1, but higher than 0.5 µm s^−1^ during most of the period of stage 2, which was in line with the characteristics of cytoskeleton‐mediated movement. The anomalous diffusion in the perinuclear region in stage 3 possibly suggested the confined movement of T‐sEVs trapped in some perinuclear structures.

### 2.7 Fate of T‐sEVs in the Perinuclear Region of ECs

**Figure 6:**
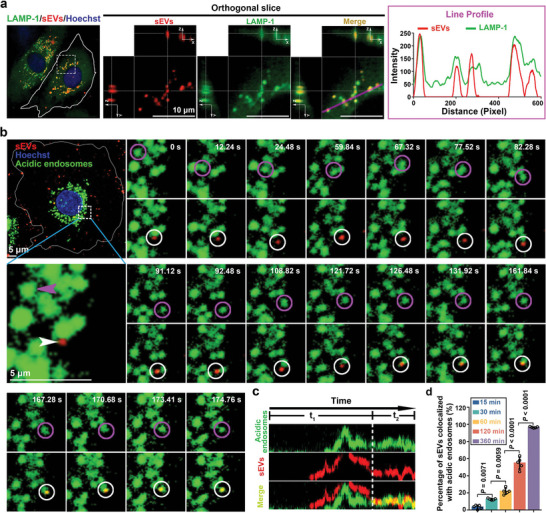
Fate of T‐sEVs in the perinuclear region of ECs. a), Identifying the destination of T‐sEVs in ECs with immunofluorescence. ECs were cocultured with QD‐labeled T‐sEVs at 37 °C for 6 h, followed by staining with various markers after fixation, the right panel was the line profile analysis which displayed the colocalization of LAMP‐1^+^ compartments (antibody, green) and T‐sEVs (QDs, red). b) Snapshots of the colocalization process between a T‐sEV (QDs, red) and an acidic endosome (Lysotracker, green) in EC as shown in Video S6 in the Supporting Information. c) Kymograph analysis showing the serial process of the contact and colocalization between a T‐sEV (red) and acidic endosomes (green). d) Quantifying the colocalization of T‐sEVs with acidic endosomes at different incubation times (15, 30, 60, 120, and 360 min). Lysotracker‐labeled ECs were cocultured with QD‐labeled T‐sEVs at 37 °C for indicated time, followed by quantification with the representative orthogonal slice image of each EC after fixation, *n* = 5, data are presented as mean ± SD, significance was determined using one‐way ANOVA followed by Tukey's multiple comparison test.

Next, we attempted to investigate the fate of T‐sEVs in the perinuclear region and to determine their final destination. We labeled different organelles in the perinuclear region with specific probes after coculture of ECs with QD‐labeled T‐sEVs. The results revealed that nearly all T‐sEVs colocalized with lysosome‐associated membrane glycoprotein 1 (LAMP‐1)+ compartments in the perinuclear region finally (**Figure** **6**a and Figure S7, Supporting Information). We then focused on tracking the dynamical movement of T‐sEVs in this region in live ECs. Results revealed that after a long‐term intermittent and relatively slow movement in the perinuclear region, T‐sEVs were beginning to contact with acidic endosomes, as positively labeled by LysoTracker, and then eventually enveloped into these organelles (*n* = 24) (Figure 6b). The kymograph analysis also demonstrated the serial process of the contact and colocalization between the T‐sEV and acidic endosome (Figure 6c). Of interest, frequent interactions between the T‐sEV and adjacent acidic endosomes suggest possible competitive or synergistic effects prior to the determination of its final destination (Video S6, Supporting Information). In addition, further analysis based on incubation time revealed that the percentage of T‐sEVs colocalized with acidic endosomes was increased over time (Figure 6d). These above results suggest that T‐sEVs interact frequently with the acidic endosomes after their arrival to the perinuclear region and can be finally enwrapped in these acidic vesicles.

### 2.8 Dissection of the miRNA Delivery and Release Behaviors of T‐sEVs in ECs

**Figure 7:**
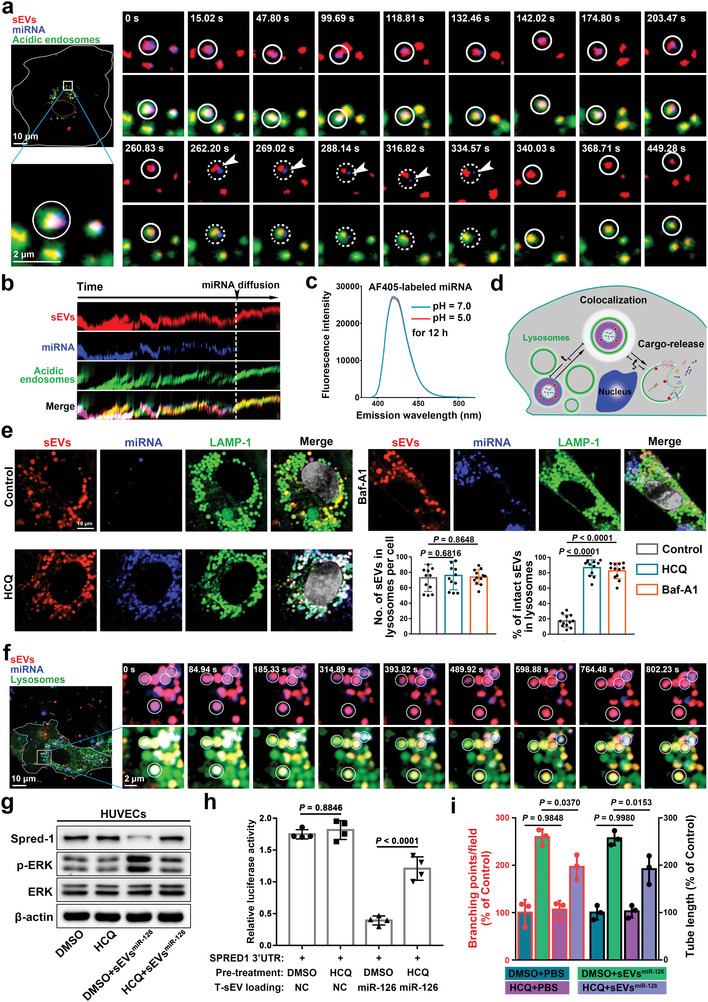
Dissection of the miRNA delivery and release behaviors of T‐sEVs in ECs. a) Snapshots of the miRNA‐release process of a dual‐color and dual‐component labeled T‐sEVs (QDs, red) shown in Video S7 in the Supporting Information. White dotted circles indicated the miRNA escape, white arrows indicated the transient retention of miRNA signal (Alexa Fluor405, blue) after the signal separation. b) The Kymograph analysis of the T‐sEV shown in (a). c) Evaluating the effects of acidic environment on the fluorescence intensity of Alexa Fluor405 (AF405)‐labeled miR‐126. AF405‐labeled miR‐126 were stored in pH = 7.0 or 5.0 PBS for 12 h, followed by the comparison of their emission spectrum. d) Schematic illustration of the miRNA‐release process of T‐sEVs in ECs. e) Exploring the effects of hydroxychloroquine sulfate (HCQ, 100 µm) and bafilomycin A1 (Baf‐A1, 10 µm) on the integrity of T‐sEVs in lysosomes (green pseudo‐color), *n*  = 12, data are presented as mean ± SD, significance was determined using two tailed unpaired *t*‐test. f) Snapshots of the stable colocalization of QD signal and miRNA signal (Alexa Fluor405, blue) in lysosomes (green pseudo‐color) shown in Video S9 in the Supporting Information. g) The activation of Spred‐1/ERK signaling in ECs after incubation with indicated T‐sEVs were detected by western blotting. ECs were pretreated with 100 µm hydroxychloroquine sulfate (HCQ) or DMSO for 1 h and cocultured with T‐sEVs which were preloaded with miR‐126. h, Dual‐luciferase reporter assay verified the functional activity of miRNA cargos released by T‐sEVs in ECs. The ECs were first transfected with pmirGLO‐SPRED1 3’UTR, then pretreated with 100 µm hydroxychloroquine sulfate (HCQ) or DMSO for 1 h and followed by coincubation with negative control mimics‐loaded T‐sEVs (negative control, NC) or miR‐126 mimics‐loaded T‐sEVs (miR‐126). All the groups were finally lysed and subjected to the luminescence scan. *n* = 4, data are presented as mean ± SD, significance was determined using one‐way ANOVA followed by Tukey's multiple comparison test. i) The effects of acidification‐promoted miR‐126‐release mediated by T‐sEVs on the angiogenic capacity of ECs were evaluated by in vitro tube formation assay, *n* = 3, data are presented as mean ± SD, significance was determined using one‐way ANOVA followed by Tukey's multiple comparison test.

The delivery of biomolecules to their recipient cells is the key event of T‐sEV‐mediated cell‐to‐cell communication, usually leading to tumor progression. Based on the above findings of T‐sEV transportation dynamics, we then aimed to dissect the cargo‐delivery behaviors of T‐sEVs in ECs. Given an increasing interest in the functionality of miRNAs carried by EVs,^[^ ^36^ ^]^ not only for pathological studies but also for RNA interference‐based therapeutics, we here focused on the miRNA delivery process of T‐sEVs. Under optimized procedure and parameters (Figure S8a–d, Supporting Information), fluorescently labeled miR‐126, a key proangiogenic miRNA enriched in T‐sEVs, were loaded into aforementioned QD‐labeled T‐sEVs with a high efficiency of 96% using electroporation, a technique that has been widely used by us and others for the functionalization of sEVs.^[^ ^22^, ^37^, ^38^ ^]^ The RNase protection assay also confirmed the successful loading of miRNA into the T‐sEV within (Figure S8e–g, Supporting Information). This multiplexed labeling strategy realized the specific labeling of sEV membrane with controllable numbers of QDs and the concurrent fluorescent labeling of their inner miRNA cargos. The multilabeled T‐sEVs were then applied to the treatment of ECs and employed for single particle tracking. As shown in **Figure** **7**a, T‐sEVs and the miRNA cargos were keeping colocalized throughout the whole transportation process, from the cell periphery to the cell nucleus. At the end of the confined movement in stage 3, we observed the colocalization of T‐sEVs (QDs 705, red), miRNA cargos (Alexa Fluor405, blue), and acidic endosomes (Lysotracker, green), suggesting that T‐sEVs and their cargos were trapped by acidic endosomes. However, the colocalization, lasting for only a several minutes, then quickly collapsed due to the signal separation of miRNAs with T‐sEVs (Figure 7a and Video S7, Supporting Information). The kymograph analysis further highlighted the process in which the T‐sEV signal maintained a high spatiotemporal synchronization with the acidic endosome signal both before and after the gradual separation of the miRNA signal (Figure 7b). The Alexa Fluor405, we used to label the luminal miRNA cargos, is a bright and stable fluorophore in the acidic context based on both our results (Figure 7c) and related reports, thus largely excluding the possibility of interpreting fluorescence quenching as signal separation. Of note, both the snapshots of the whole interaction process and the kymograph analysis recorded a transient stay of miRNA signals near the outside of acidic endosome during the signal separation process. These results implied that the cargos carried in T‐sEVs were probably released into the perinuclear region through a lysosome‐mediated manner (Figure 7d), likely the acidification‐promoted membrane fusion. Therefore, we applied fluorescent protein to develop a pH‐independent strategy to study the role of acidification in the cargo‐release dynamic. The lysosomes in ECs were lighted by mCherry‐labeled LAMP‐1 (Figure S9a, Supporting Information). Nearly all T‐sEVs were colocalized with LAMP‐1^+^ lysosomes in ECs after 6 h (Figure S9b, Supporting Information). More than that, we also recorded the typical signal separation phenomenon during the miRNA‐release process of T‐sEVs in lysosomes (Figure S9c and Video S8, Supporting Information). Those repeatable results not only validated our hypothesis about miRNA‐release dynamics of T‐sEVs in ECs but also confirmed the reliability of our system. To further determine the role of acidification on the cargo‐release dynamics, ECs were cocultured with T‐sEVs in the presence of hydroxychloroquine (HCQ) or Bafilomycin A1 (Baf‐A1), two specific inhibitors of endosomal/lysosomal acidification.^[^ ^39^, ^40^ ^]^ The results indicated that most T‐sEVs released their cargos at 6 h under normal condition, while disrupting the acidification of lysosomes significantly suspended the cargo‐release process of T‐sEVs (Figure 7e). The results of real‐time tracking further demonstrated that HCQ treatment disrupted the trigger of cargo‐release, as evidenced by the stable colocalization of QD signal and miRNA signal in lysosomes (Figure 7f and Video S9, Supporting Information). The functional significance of lysosome for T‐sEV‐exerted miRNA delivery and biological effects in their recipient ECs were also studied. The results revealed that miR‐126‐loaded T‐sEVs effectively decreased the expression level of its target protein Spred‐1 in ECs, thereby leading to an abnormal activation of the extracellular regulated protein kinase (ERK) signaling pathway (Figure 7g). Dual‐Luciferase reporter assay further demonstrated that this effect was dependent on the direct regulation of Spred‐1 mRNA stability by those delivered miR‐126 (Figure 7h and Figure S10, Supporting Information). As a result, the tube formation of ECs was markedly increased, suggesting enhanced angiogenic activities (Figure 7i). However, treatment with HCQ nearly abolished the effects of miR‐126‐loaded T‐sEVs on the Spred‐1/ERK pathway (Figure 7g,h) and significantly attenuated their proangiogenic effects on ECs (Figure 7i and Figure S11a,b, Supporting Information), suggesting the importance of the lysosome acidification in T‐sEV‐exerted miRNA delivery and angiogenic function. Together, these above results not only provide the first detailed spatiotemporal dynamics of T‐sEV‐exerted miRNA release in recipient ECs but also highlight the potential strategy to target this process for therapeutic purposes.

EVs are highly heterogeneous populations of bilayer‐enclosed structures secreted by a variety of cells.^[^ ^1^ ^]^ The nanosized sEVs, including exosomes and other membrane vesicles with diameters below 200 nm, are currently the most studied subpopulation of EVs, owing to their distinct biogenesis mechanisms, physicochemical properties, and physiopathological functions.^[^ ^3^, ^41^ ^]^ The discovery of sEVs inaugurates a prosperous research field that offers novel mechanisms for intercellular communication and promising sources for natural theranostic vectors.^[^ ^2^, ^42^ ^]^ For a better understanding of the biological functions and potential applications, large numbers of studies have been dedicated to the characterization of sEV‐mediated cell–cell communication, with a particular focus on their cargo delivery behaviors.^[^ ^43^, ^44^ ^]^

One of the most efficient approach to this end is the real‐time visualization based on high‐resolution imaging, especially the single particle tracking technique.^[^ ^19^, ^45^ ^]^ Different from traditional cellular imaging technologies, which interpret the characteristics of physiological activities based on average information of multiple targets intercepted from random snapshots, single particle tracking is a powerful strategy to study the dynamic process of biological events involving heterogeneous nanosized individuals,^[^ ^19^, ^45^ ^]^ just like sEVs. Indeed, the rapid progress in imaging equipment has made it possible for monitoring biological processes at a single particle or even a single molecule level in situ.^[^ ^9^, ^46^ ^]^ Notably, several previous studies have attempted to investigate the dynamic process of sEV‐mediated intercellular communication.^[^ ^47^, ^48^, ^49^ ^]^ However, these studies are still a long way from the long‐term tracking of sEVs in real time, thereby hesitating the acquirement of accurate and detailed dynamics. This is mainly due to the major defects in the current labeling strategy for sEVs, which is actually of particular importance for high‐quality imaging and tracking. In reference to the traditional labeling for cells, fluorescent proteins and organic fluorescent dyes have been most employed to label and visualize sEVs in the previous studies. However, either fluorescent proteins or organic dyes have their own limitation in the real‐time and long‐term tracking of sEVs.^[^ ^8^ ^]^ In contrast to the traditional labeling materials, QDs are a new class of nanosized probe for real‐time bioimaging and single particle tracking due to their outstanding brightness, superior photobleaching resistance, and tunable emission wavelengths.^[^ ^18^, ^20^ ^]^ Some studies, including ours, visualized EVs based on different QD‐based labeling strategies, but failed to elucidate the delicate steps of EV‐mediated intercellular communication.^[^ ^21^, ^22^, ^23^, ^24^ ^]^ Given the unique advantages of QDs for single particle tracking and also the importance of single particle tracking to the study of sEVs, our present study proposed a reliable and controllable strategy for the membrane specific and quantitative labeling of sEVs with QDs, and then further developed a QD‐based SPT technique to dissect the spatiotemporal dynamics of sEVs’ behaviors in their recipient cells. Depending on this QD‐based single particle tracking platform, the entire dynamic process of T‐sEV transportation and cargo‐delivery in the recipient ECs were successfully tracked in real time and analyzed in detail. However, some blemish still could be questioned for this QD‐based labeling strategy at present. First, the membrane biotinylation strategy labeled a general population of EVs, which would be not applicative for the purpose to only focus on a particular EV subtype. Second, the longevity of the association of the QD with the EVs should be studied in deep, such as by using our SPT platform. In spite of this, the excellent performance of our biotinylation‐based labeling strategy in vitro and in vivo still highlighted its potential value.

It is believed that the uptake of sEVs by recipient cells is the first critical step to deliver their cargos and exert corresponding biological functions.^[^ ^11^, ^50^ ^]^ Previous studies have suggested that sEVs might be taken up by their recipient cells by either membrane fusion or endocytosis, being attributed to the heterogeneity in both sEV subpopulations and recipient cell types.^[^ ^11^, ^50^ ^]^ In this study, we determined an endocytosis‐dependent entry of T‐sEVs into the ECs, as evidenced by the efficient and persistent colocalization of sEVs and endocytic vesicles. The active energy‐consuming feature and results based on specific inhibitors and dynamic‐tracking further revealed an essential role of clathrin‐mediated endocytosis in the cellular entry of T‐sEVs. Of note, the intrinsic blinking behavior of a single QD, also known as fluorescence intermittency,^[^ ^51^ ^]^ would further cause transient fluorescence quenching following the sudden attenuation of target signal upon membrane fusion‐induced redistribution of QDs on the cytomembrane. Thus, the persistent and strong signals of QD‐labeled sEVs around the cytomembrane of ECs also helped to distinguish endocytosis from membrane fusion in a much easier way.

Following the endocytosis‐dependent entry into the cell, we witnessed the entire trafficking of sEVs from the cell periphery to the cell nucleus. Of note, an early study, using fluorescent dye as the labeling material, has also observed a directed trafficking of T‐sEVs from the cell surface to the perinuclear region.^[^ ^52^ ^]^ But this study failed to characterize more detailed dynamics during this process due to the drawbacks in labeling strategy, which severely limited imaging speed and tracking period. Thus, the authors encouraged the development of better labeling strategy to achieve high‐speed imaging and long‐term tracking. In the present study, by taking advantage of the superior optical properties of QD‐labeled T‐sEVs endowed by our developed labeling strategy, we realized the long‐term tracking of T‐sEVs with fast imaging rate. The fast imaging speed helped to reconstruct the trajectories of each individual sEV more realistically, thus better fitting the instantaneous velocities for accurate identification of transportation patterns. As a result, we revealed a striking three‐stage transportation pattern of sEVs in ECs and more importantly characterized the spatiotemporal dynamics of each stage in detail. Stage 1 was a microfilament‐based slow directed motion in the cell periphery region. Stage 2 was a long‐distance microtubule‐based fast directed motion from the cell periphery to the cell nucleus. Stage 3 was a slow confined movement in the perinuclear region. Since the elaborated molecular mechanisms behind the cytoskeleton‐mediated transportation of T‐sEVs are currently unclear, it is a fascinating direction to perform in‐depth mechanistic studies using the QD‐based single particle tracking platform.

The final and also the most important step in sEV‐mediated intercellular interaction is the cargo release, which may potentially induce phenotypic and functional changes in the recipient cells.^[^ ^33^, ^53^ ^]^ Although this step has received great attention recently, the dynamic cargo‐release behavior of sEVs has been sparely visualized, severely hindering the dissection of underlying spatiotemporal dynamics. Extensive studies have demonstrated the significant importance of sEV‐delivered miRNAs, especially to the benefit of tumor progression,^[^ ^36^, ^54^, ^55^ ^]^ we therefore selected miR‐126, an angiogenesis driver enriched in T‐sEVs,^[^ ^56^, ^57^ ^]^ as the model cargo to study the cargo‐release behaviors of T‐sEVs in ECs. Since in situ labeling endogenous miRNAs carried by sEVs still faces major hurdles, we loaded exogenous fluorescently labeled miR‐126 into QD‐labeled T‐sEVs by electroporation, thus achieving dual‐color and dual‐component labeling of T‐sEVs. By using these dual‐labeled T‐sEVs for single particle tracking, we visualized and dissected the dynamic process of T‐sEV‐exerted miRNA release in ECs. It was uncovered that, following a long‐time confined motion in the perinuclear region, T‐sEVs began to fused with the lysosomes for their release of the carried miRNA, as demonstrated by the gradual signal separation of T‐sEV membrane and luminal miRNA cargos after their colocalization with lysosomes. Our results, based on both dynamic tracking and functional analysis, suggested that the luminal miRNA cargos were likely to be functional delivery rather than degraded. Importantly, it was revealed that the miRNA‐release by T‐sEVs after their fusion with the lysosomes is a quite fast process, which was mostly completed in 10 s. Those findings may partially explain the inadequacy of traditional methods in tracking and dissecting this dynamic process. The successful visualization and tacking of the miRNA‐release process may be mainly attributed to our biotinylation‐based membrane‐specific labeling strategy that possesses outstanding specificity to biotinylated T‐sEVs (not other nonbiotinylated organelles in the recipient cell) and maximizes the optical properties of QDs. The direct witness of the signal separation of T‐sEV and its carried miRNA near acidic endosomes indicated that the cargo‐release by T‐sEVs is possibly through acidic pH‐triggered membrane fusion.^[^ ^48^ ^]^ In support of this hypothesis, blocking endosome acidification by HCQ significantly reduced T‐sEV‐released miR‐126 into ECs and accordingly impaired the downstream angiogenic effects. Of note, several previous studies based on traditional static colocalization experiments have implicated a link between internalized sEVs and nuclear envelope.^[^ ^58^, ^59^ ^]^ However, our long‐time dynamic tracking of hundreds of individual sEV particles suggested the lysosomes as the potential destination for T‐sEV‐mediated cargo delivery in ECs. This discrepancy, possibly resulted from the difference in research methods, highlights the importance of single particle tracking to the study of sEV‐related dynamic process. Another study, using CD63‐green fluorescent protein (GFP) single‐labeled sEVs, found the stop‐and‐go movement of sEVs along the endoplasmic reticulum and raise the possibility of cargo‐release by sEVs in endoplasmic reticulum.^[^ ^60^ ^]^ Our study based on the dual‐labeled sEVs, for both their membrane and inner miRNA, confirmed the miRNA‐release following the loss of integrity of T‐sEVs in lysosomes. This emphasizes that, in addition to the dynamic single particle tracking, multicolor and multicomponent labeling is also critical to understanding the complex dynamics in sEV‐mediated intercellular communication. Although we demonstrated the pivotal role of lysosomes in T‐sEV‐mediated miRNA‐delivery in ECs, the heterogeneity in sEVs and target cells still queried whether these are universal characteristics underlying sEV‐mediated intercellular communication. The QD‐based single particle tracking platform developed here will help to answer this question by serving as a standardized and general tool for future in‐depth studies about the dynamic interplays between different sEVs and their diverse target cells.

## 3 Conclusion

In summary, by virtue of an advanced membrane‐specific labeling strategy using QDs, we provided a versatile QD‐based single particle tracking platform for studying the dynamic behaviors of sEVs in their recipient cells in real time and long term. It was dissected, for the first time, that most T‐sEVs enter into ECs via clathrin‐dependent endocytosis, then move slowly in the cell periphery, then converge rapidly to the cell nucleus, then confinedly move in the perinuclear region, and finally interact and fuse with the target endosomes for miRNA release, thus presenting a vivid multistage pattern for T‐sEV transportation and cargo delivery into the target cells (Figure S11, Supporting Information). Although we have integrated the current most advanced tracking strategies and the most classical analytical methods to dissect the EV‐mediated intercellular communication, there may still be many complex and heterogeneous details lurking behind the scenes. Our research is just one more small step in the journey of getting infinitely closer to the fact. The findings by this study help to answer the long‐pending question about how sEVs transport and deliver cargos into their recipient cells and may also provide important clues for future development of novel therapeutic strategies against sEV‐mediated intercellular communication.

## 4 Experimental Section

### Cell Culture

The human oral squamous cell line CAL27 cells were cultured in Dulbecco's modified Eagle's medium (Gibco) supplemented with 10% fetal bovine serum (FBS, PAN Biotech). HUVECs were cultured in endothelial cell basal medium (ECM, ScienceCell) supplemented with 10% FBS and 1% endothelial cell growth supplement (ECGS). The human monocyte line THP‐1 cells were passaged every 3 d in Roswell Park Memorial Institute (RPMI) 1640 (Gibco) medium supplemented with 10% FBS (PAN Biotech). For the biotinylation, cells were cultured and passaged in complete growth medium containing DSPE‐PEG‐Biotin (0–30 µg mL^−1^) at 37 °C with 5% CO_2_ in a humidified atmosphere for indicated time.

### Isolation and Labeling of EVs

For purification of cell‐derived EVs, biotinylated cells were cultured in aforementioned medium except the supplemented FBS were ultracentrifuged at 120 000 × *g* overnight in advance to deplete the EVs. After 48 h cell culture, supernatants were collected and centrifuged at 2000 × *g* for 20 min twice to remove dead cells or cell debris. Total EVs were collected by centrifuging at 120 000 × *g*, 4 °C for 70 min (Beckman Coulter, Optima XPN‐100) and suspended in PBS (Hyclone). For sEV isolation, the cell debris‐free supernatants were then centrifuged at 16 500 × *g* for 45 min at 4 °C and recentrifuged at 120 000 × *g* for 70 min (Beckman Coulter, Optima XPN‐100). The pelleted sEVs were suspended in PBS and purified by another ultracentrifugation at 120 000 × *g* for 2 h. For fluorescence imaging, sEVs were incubated with CellMask Green (Invitrogen) or PKH26 (Sigma) according to the manufacture's instruction. After incubation, the mixture was diluted with PBS and centrifuged at 120 000 × *g* for 40 min (Beckman Coulter, Optima MAX‐XP) to remove excess free dye and retrieve the dye‐labeled sEVs. For QD‐based labeling, biotinylated sEVs were incubated with SA‐QDs 705 (Invitrogen) at 37 °C for 20 min at the indicated concentration. The mixture was overlaid on a sucrose gradient (0.1, 0.5, 1.0, 1.5, 2, and 2.5 M) in an SW41Ti tube. After ultracentrifugation at 180 000 × g for 12 h at 4 °C (Beckman Coulter, Optima XPN‐100), 12 fractions of 1 mL were collected and diluted with 30 mL PBS followed by recentrifuging at 120 000 × *g* for 70 min (Beckman Coulter, Optima XPN‐100) to retrieve the pellets.

### Western Blot Analysis

25 µg denatured cell lysates or 4 × 10^10^ sEVs were separated on 10% sodium dodecyl sulfate polyacrylamide gel electrophoresis (SDS–PAGE). Following electrophoresis, proteins were electroblotted to polyvinylidene fluoride membranes (Roche Applied Science). Then, the blots were blocked with 5% nonfat milk and incubated overnight at 4 °C with primary antibodies. The dilution of antibodies was determined based on the suppliers’ recommendation and results of preliminary experiments. The next day, the blots were incubated with corresponding secondary antibodies conjugated with horseradish peroxidase (HRP) (Jackson ImmunoResearch) at room temperature for 1 h. Finally, the immunoblots were visualized by using enhanced chemiluminescence (ECL) detection reagents (Pierce) and recorded with chemiluminescence imaging system (Sage Creation). *β*‐actin and Ponceau S were used as loading control. CD63, CD9, and Alix were used as markers of sEVs. Calnexin and GM130 were used to validate the potential contamination of sEVs with cell debris.

### RNA Isolation and quantitative Polymerase Chain Reaction (qPCR) Analysis

Total RNA of sEVs were extracted with the miRNeasy Kit (QIAGEN) according to the manufacturer's protocol. Following the synthesis of complementary DNA with 250 ng total RNA, real‐time PCR was performed by using Mir‐X miRNA qRT‐PCR TB Green Kit (Takara) on a QuantStudio 6 Flex Real‐time Polymerase Chain Reaction (PCR) System (Applied Biosystems) under standard conditions. U‐6 was used as internal control. All microRNA primers were purchased from GeneCopoeia. The fold changes of different miRNAs were calculated with the comparative threshold cycle method.

### TEM

QD‐labeled sEVs or free SA‐QDs suspended in PBS were dropped on formvar carbon‐coated nickel grids. Following negative staining with 2% uranyl acetate, the grids were air‐dried. The results were recorded using a JEM‐1011 transmission electron microscope (Hitachi, Japan).

### NTA

The size distribution and concentration of sEVs were analyzed using ZetaView Nanoparticle Tracking Analyzer (Particle Metrix, Germany). The quality check of sample cell and focus of instrument were first performed after switching on the power. Then, the system was calibrated with 100 nm polystyrene standard nanobeads. Samples were diluted in PBS to reach appropriate final concentrations and loaded into the cell at a constant rate. The measurement para meters of different groups were kept consistent (camera sensitivity = 80, shutter speed = 63, frame rate = 7.5). For each sample, the video was acquired from eleven different positions of the cell and the reading of each positions was repeated two times. Measurement data from the ZetaView were analyzed using the supporting automated software (ZetaView 8.05.11).

### Electroporation

The loading of fluorophore‐labeled miRNA mimics into sEVs were realized using the Bio‐Rad Gene Pulser Xcell Electroporation System. sEVs (20 µg) and indicated concentrations of Alexa Fluor 405‐ or carboxyfluorescein (FAM)‐labeled microRNA mimics were added into 400 µL electroporation buffer (Bio‐Rad) in 0.4 cm cuvettes (Bio‐Rad). Electroporations were performed with parameter setting at 250 V and 350 µF for the indicated times of repetition. Then, the mixture was incubated at 37 °C for 20 min and centrifuged at 120 000 × *g* for 70 min in PBS to remove the excess free mimics.

### Flow Cytometry Analysis

The biotinylation efficiency of EVs and the optimization of electroporation were analyzed by using an Apogee A‐50 Micro Flow Cytometer (Apogee Flow Systems) equipped with 375, 405, 488, and 638 nm lasers. The reference beads (ApogeeMix, Apogee Flow Systems) composed of a mixture of nonfluorescent silica beads (Si, refractive index ≈1.42) with diameters of 180, 240, 300, 590, 880, 1300, and 110 nm, 500 nm green fluorescent latex spheres (polystyrene, refractive index ≈1.59) were used to set the thresholds for light scattering and help to gate sEVs and lEVs. The tubing was washed with double‐distilled water after each sampling. Analysis was performed at a flow rate of 1.5 µL min^−1^ using a 150 µL sample volume for at least total 3 × 10^5^ particles.

### Fluorescence Imaging

The cells were seeded on the glass bottom of dedicated confocal dishes (NEST) and cultured for 24 h. Hoechst 33342 (Sigma) and CellMask Green Plasma Membrane Stain (Invitrogen), whose fluorescent excitation/emission maxima were 346/460 nm and 522/535 nm separately, were used to label cell nucleus and the plasma membrane of ECs. The working solutions with indicated final concentrations of 5‐(*N*‐ethyl‐*N*‐isopropyl) amiloride (Selleckchem), CPZ HCl (Selleckchem), methyl‐*β*‐cyclodextrin (M*β*CD, Selleckchem), cytochalasin D (Cyto D, Selleckchem), and NOC (Selleckchem) were prepared by diluting the stock solutions with ECM. ECs were pretreated with inhibitor at 37 °C for 1 h before the attachment of sEVs and the corresponding inhibitor was maintained throughout the preparation stage. For static imaging, ECs were incubated with labeled sEVs (30 µg mL^−1^) at indicated temperature for indicated time. Then, the dish was washed gently by PBS for three times and fixed with 4% paraformaldehyde solution for 15 min at room temperature. Before observation, the dish was washed gently for three times with PBS. For live cell tracking, 50 nK LysoTracker Green (Meiunbio) was used to specifically label acidic endosomes according to research purpose. ECs were then incubated with labeled sEVs (30 µg mL^−1^) at 37 °C for 10 min. After washed gently with PBS, the dish was immobilized in an online live cell culture chamber (INUBG2‐PI, TOKAI HIT). Time‐lapse live‐cell images were acquired with a spinning‐disk confocal microscope system (the Andor Revolution XD) which was equipped with an invert microscope (IX 81, Olympus). A Nipkow disk type confocal unit (CSU 22, Yokogawa), an Emission filter wheel (Sutter Instruments), and an electron multiplying charge‐coupled device (EMCCD) (Andor iXon Ultra 897) were equipped to support the imaging speed. Diode pumped solid state (DPSS) lasers at 405, 488, 561, and 640 nm were used to excite Hochest 33342/Alexa Fluor 405, CellMask Green/FAM/LysoTracker Green, PKH26, and QDs 705, respectively.

### Image Analysis

The trajectories of sEVs were reconstructed from the original video stack with Imaging‐Pro‐Plus software (Media Cybernetics) by aligning the proximity and similarity in coordinate and intensity of each spots representing sEVs or corresponding structures in every frame. Only the trajectories within the focal plane were processed for quantitative analysis of single particle tracking. The MSD was calculated by the user‐written program with MATLAB software (MathWorks). The different motion modes were determined by fitting the dependence of MSD against time interval (Δ*t*) with the formulas as below: MSD = 4*D*Δ*t* + constant (free diffusion), MSD = 4*D*Δ*t* ^*α*^ + constant (restricted diffusion) and MSD = 4*D*Δ*t* + (*V*Δ*t*)^2^ + constant (directed diffusion), where *D* and *V* represents the diffusion coefficient and the mean velocity respectively. The orthogonal slice view was obtained by UltraVIEW (PerkinElmer) to display the relative position of particles and cell in a 3D view. Kymograph analysis was performed with Image J. Line profile of colocalization or endocytosis were exhibited by using Imaging‐Pro Plus software.

### Wound Healing Assay

ECs were seeded in a six‐well plate and cultured in 5% FBS ECM supplemented with HCQ or equal dimethylsulfoxide (DMSO), and sEVs were added 1 h later for coculture. When grown to 90% confluence, the monolayer of ECs was scraped with the tip of a sterile micropipette. After rinsed with PBS, the cells were cultured under the same condition as before for 24 h. The area in the gap were recorded under a phase microscope (Nikon) and measured with Image J.

### Boyden Chamber Assay

ECs were cultured in 5% FBS ECM supplemented with HCQ or equal DMSO, and sEVs were added 1 h later. After 36 h, 5 × 10^4^ cells suspend in ECM were seeded into the upper chamber, and 800 µL of ECM supplemented with 3% FBS and 1% ECGS were added to the bottom chamber. After fixation and staining, the migrated cells on the lower surface of the filter were observed and captured under a bright field microscope (Leica).

### Tube Formation Assay

ECs were pretreated with HCQ or DMSO under normal conditions for 1 h, and sEVs were then added to the medium to indicated concentrations. After 24 h of culture, cells from each group were separately digested and seeded into 24‐well plate (8 × 10^4^ cells per well) which were precoated with Matrigel (BD Biosciences). The cells were kept cultured under normal conditions and the capillary‐like structures were recorded under a phase microscope (Nikon). The branching points and total tube length were quantified by angiogenesis analyzer plugin of Image J.

### Statistical Analysis

Data were analyzed for statistical significance using GraphPad Prism v.7.0. The sample size (*n*) for every experiment is noted in each figure legend. Two‐tailed paired or unpaired student's *t*‐test was used to compare the difference between two groups and one‐way analysis of variance (ANOVA) followed by Tukey's multiple comparison test were performed to compare the differences between multiple groups, or as indicated in the figure legend. Data are presented as mean ± standard deviation (SD).

## Conflict of Interest

The authors declare no conflict of interest.

## Supporting information

Supporting Information

Supplemental Video 1

Supplemental Video 2

Supplemental Video 3

Supplemental Video 4

Supplemental Video 5

Supplemental Video 6

Supplemental Video 7

Supplemental Video 8

Supplemental Video 9

## Data Availability

The data that support the findings of this study are available from the corresponding author upon reasonable request.

## References

[1] G. Raposo , W. Stoorvogel , J. Cell Biol. 2013, 200, 373. 23420871 10.1083/jcb.201211138 PMC3575529

[2] M. Tkach , C. Thery , Cell 2016, 164, 1226. 26967288 10.1016/j.cell.2016.01.043

[3] R. Kalluri , V. S. LeBleu , Science 2020, 367, eaau6977. 32029601 10.1126/science.aau6977 PMC7717626

[4] S. L. N. Maas , X. O. Breakefield , A. M. Weaver , Trends Cell Biol. 2017, 27, 172. 27979573 10.1016/j.tcb.2016.11.003 PMC5318253

[5] M. Piffoux , A. Nicolas‐Boluda , V. Mulens‐Arias , S. Richard , G. Rahmi , F. Gazeau , C. Wilhelm , A. K. A. Silva , Adv. Drug Delivery Rev. 2019, 138, 247. 10.1016/j.addr.2018.12.009 30553953

[6] S. E. Andaloussi , I. Mager , X. O. Breakefield , M. J. Wood , Nat. Rev. Drug Discovery 2013, 12, 347. 23584393 10.1038/nrd3978

[7] M. S. Panagopoulou , A. W. Wark , D. J. S. Birch , C. D. Gregory , J. Extracell. Vesicles 2020, 9, 1710020. 32002172 10.1080/20013078.2019.1710020 PMC6968689

[8] S. T. Chuo , J. C. Chien , C. P. Lai , J. Biomed. Sci. 2018, 25, 91. 30580764 10.1186/s12929-018-0494-5 PMC6304785

[9] B. Huang , M. Bates , X. Zhuang , Annu. Rev. Biochem. 2009, 78, 993. 19489737 10.1146/annurev.biochem.77.061906.092014 PMC2835776

[10] Y. Ma , X. Wang , H. Liu , L. Wei , L. Xiao , Anal. Bioanal. Chem. 2019, 411, 4445. 30790020 10.1007/s00216-019-01638-z

[11] M. Mathieu , L. Martin‐Jaular , G. Lavieu , C. Thery , Nat. Cell Biol. 2019, 21, 9. 30602770 10.1038/s41556-018-0250-9

[12] P. Carpintero‐Fernandez , J. Fafian‐Labora , A. O'Loghlen , Front. Mol. Biosci. 2017, 4, 79. 29234666 10.3389/fmolb.2017.00079 PMC5712308

[13] G. Corso , W. Heusermann , D. Trojer , A. Görgens , E. Steib , J. Voshol , A. Graff , C. Genoud , Y. Lee , J. Hean , J. Z. Nordin , O. P. B. Wiklander , S. El Andaloussi , N. Meisner‐Kober , J. Extracell. Vesicles 2019, 8, 1663043. 31579435 10.1080/20013078.2019.1663043 PMC6758720

[14] S. H. Ibrahim , P. Hirsova , K. Tomita , S. F. Bronk , N. W. Werneburg , S. A. Harrison , V. S. Goodfellow , H. Malhi , G. J. Gores , Hepatology 2016, 63, 731. 26406121 10.1002/hep.28252 PMC4764421

[15] P. Puzar Dominkus , M. Stenovec , S. Sitar , E. Lasic , R. Zorec , A. Plemenitas , E. Zagar , M. Kreft , M. Lenassi , Biochim Biophys Acta Biomembr 2018, 1860, 1350. 29551275 10.1016/j.bbamem.2018.03.013

[16] F. Ender , P. Zamzow , N. V. Bubnoff , F. Gieseler , Int. J. Mol. Sci. 2019, 21, 291. 31906247 10.3390/ijms21010291 PMC6981603

[17] X. Michalet , F. F. Pinaud , L. A. Bentolila , J. M. Tsay , S. Doose , J. J. Li , G. Sundaresan , A. M. Wu , S. S. Gambhir , S. Weiss , Science 2005, 307, 538. 15681376 10.1126/science.1104274 PMC1201471

[18] K. D. Wegner , N. Hildebrandt , Chem. Soc. Rev. 2015, 44, 4792. 25777768 10.1039/c4cs00532e

[19] H. Shen , L. J. Tauzin , R. Baiyasi , W. Wang , N. Moringo , B. Shuang , C. F. Landes , Chem. Rev. 2017, 117, 7331. 28520419 10.1021/acs.chemrev.6b00815

[20] N. Kaji , M. Tokeshi , Y. Baba , Chem. Rec. 2007, 7, 295. 17924442 10.1002/tcr.20128

[21] G. Chen , J. Y. Zhu , Z. L. Zhang , W. Zhang , J. G. Ren , M. Wu , Z. Y. Hong , C. Lv , D. W. Pang , Y. F. Zhao , Angew. Chem., Int. Ed. Engl. 2015, 54, 1036. 25412570 10.1002/anie.201410223

[22] J. Y. Zhao , G. Chen , Y.‐P. Gu , R. Cui , Z. L. Zhang , Z. L. Yu , B. Tang , Y. F. Zhao , D. W. Pang , J. Am. Chem. Soc. 2016, 138, 1893. 26804745 10.1021/jacs.5b10340

[23] Z. L. Yu , W. Zhang , J. Y. Zhao , W. Q. Zhong , J. G. Ren , M. Wu , Z. L. Zhang , D. W. Pang , Y. F. Zhao , G. Chen , Adv. Funct. Mater. 2017, 27, 1603524.28824357

[24] E. Bonsergent , G. Lavieu , FEBS Lett. 2019, 593, 1983. 31175663 10.1002/1873-3468.13472

[25] T. Yamamoto , Y. Teramura , T. Itagaki , Y. Arima , H. Iwata , Sci. Technol. Adv. Mater. 2016, 17, 677. 27877914 10.1080/14686996.2016.1240006 PMC5101893

[26] Y. Wan , G. Cheng , X. Liu , S. J. Hao , M. Nisic , C. D. Zhu , Y. Q. Xia , W. Q. Li , Z. G. Wang , W. L. Zhang , S. J. Rice , A. Sebastian , I. Albert , C. P. Belani , S. Y. Zheng , Nat. Biomed. Eng. 2017, 1, 0058. 28966872 10.1038/s41551-017-0058 PMC5618714

[27] L. Chen , S. Mou , F. Li , Y. Zeng , Y. Sun , R. E. Horch , W. Wei , Z. Wang , J. Sun , ACS Appl. Mater. Interfaces 2019, 11, 46183. 31718127 10.1021/acsami.9b17015

[28] C. P. Lai , O. Mardini , M. Ericsson , S. Prabhakar , C. Maguire , J. W. Chen , B. A. Tannous , X. O. Breakefield , ACS Nano 2014, 8, 483. 24383518 10.1021/nn404945r PMC3934350

[29] D. Dong , L. Zhu , J. Hu , D. W. Pang , Z. L. Zhang , Talanta 2019, 200, 408. 31036202 10.1016/j.talanta.2019.03.069

[30] V. Pospichalova , J. Svoboda , Z. Dave , A. Kotrbova , K. Kaiser , D. Klemova , L. Ilkovics , A. Hampl , I. Crha , E. Jandakova , L. Minar , V. Weinberger , V. Bryja , J. Extracell. Vesicles 2015, 4, 25530. 25833224 10.3402/jev.v4.25530 PMC4382613

[31] J. H. T. Luong , S. K. Vashist , ACS Omega 2020, 5, 10. 31956746 10.1021/acsomega.9b03013 PMC6963918

[32] S. Han , W. Y. Shih , W. H. Shih , ChemistrySelect 2017, 2, 7332. 30410961 10.1002/slct.201700855 PMC6219619

[33] S. Maacha , A. A. Bhat , L. Jimenez , A. Raza , M. Haris , S. Uddin , J. C. Grivel , Mol. Cancer 2019, 18, 55. 30925923 10.1186/s12943-019-0965-7 PMC6441157

[34] M. Lakadamyali , M. J. Rust , H. P. Babcock , X. Zhuang , Proc. Natl. Acad. Sci. USA 2003, 100, 9280. 12883000 10.1073/pnas.0832269100 PMC170909

[35] S. L. Liu , L. J. Zhang , Z. G. Wang , Z. L. Zhang , Q. M. Wu , E. Z. Sun , Y. B. Shi , D. W. Pang , Anal. Chem. 2014, 86, 3902. 24678700 10.1021/ac500640u PMC4004192

[36] M. A. Mori , R. G. Ludwig , R. Garcia‐Martin , B. B. Brandao , C. R. Kahn , Cell Metab. 2019, 30, 656. 31447320 10.1016/j.cmet.2019.07.011 PMC6774861

[37] F. Chen , J. Chen , L. Yang , J. Liu , X. Zhang , Y. Zhang , Q. Tu , D. Yin , D. Lin , P. P. Wong , D. Huang , Y. Xing , J. Zhao , M. Li , Q. Liu , F. Su , S. Su , E. Song , Nat. Cell Biol. 2019, 21, 498. 30936474 10.1038/s41556-019-0299-0

[38] S. Ramanathan , B. B. Shenoda , Z. Lin , G. M. Alexander , A. Huppert , A. Sacan , S. K. Ajit , J. Extracell. Vesicles 2019, 8, 1650595. 31489147 10.1080/20013078.2019.1650595 PMC6713176

[39] E. Boucrot , A. P. Ferreira , L. Almeida‐Souza , S. Debard , Y. Vallis , G. Howard , L. Bertot , N. Sauvonnet , H. T. McMahon , Nature 2015, 517, 460. 25517094 10.1038/nature14067

[40] D. S. D'Astolfo , R. J. Pagliero , A. Pras , W. R. Karthaus , H. Clevers , V. Prasad , R. J. Lebbink , H. Rehmann , N. Geijsen , Cell 2015, 161, 674. 25910214 10.1016/j.cell.2015.03.028

[41] A. Matsumoto , Y. Takahashi , H. Y. Chang , Y. W. Wu , A. Yamamoto , Y. Ishihama , Y. Takakura , J Extracell Vesicles 2020, 9, 1696517. 31807238 10.1080/20013078.2019.1696517 PMC6882433

[42] B. Yang , Y. Chen , J. Shi , Adv. Mater. 2019, 31, 1802896. 10.1002/adma.201802896 30126052

[43] C. Crewe , N. Joffin , J. M. Rutkowski , M. Kim , F. Zhang , D. A. Towler , R. Gordillo , P. E. Scherer , Cell 2018, 175, 695. 30293865 10.1016/j.cell.2018.09.005 PMC6195477

[44] E. Hergenreider , S. Heydt , K. Treguer , T. Boettger , A. J. Horrevoets , A. M. Zeiher , M. P. Scheffer , A. S. Frangakis , X. Yin , M. Mayr , T. Braun , C. Urbich , R. A. Boon , S. Dimmeler , Nat. Cell Biol. 2012, 14, 249. 22327366 10.1038/ncb2441

[45] K. Jaqaman , D. Loerke , M. Mettlen , H. Kuwata , S. Grinstein , S. L. Schmid , G. Danuser , Nat. Methods 2008, 5, 695. 18641657 10.1038/nmeth.1237 PMC2747604

[46] R. Strack , Nat. Methods 2019, 16, 455. 31147638 10.1038/s41592-019-0438-3

[47] M. Kanada , M. H. Bachmann , J. W. Hardy , D. O. Frimannson , L. Bronsart , A. Wang , M. D. Sylvester , T. L. Schmidt , R. L. Kaspar , M. J. Butte , A. C. Matin , C. H. Contag , Proc. Natl. Acad. Sci. USA 2015, 112, E1433. 25713383 10.1073/pnas.1418401112 PMC4378439

[48] I. Parolini , C. Federici , C. Raggi , L. Lugini , S. Palleschi , A. De Milito , C. Coscia , E. Iessi , M. Logozzi , A. Molinari , M. Colone , M. Tatti , M. Sargiacomo , S. Fais , J. Biol. Chem. 2009, 284, 34211. 19801663 10.1074/jbc.M109.041152 PMC2797191

[49] Y. Ofir‐Birin , P. Abou Karam , A. Rudik , T. Giladi , Z. Porat , N. Regev‐Rudzki , Front. Immunol. 2018, 9, 1011. 29881375 10.3389/fimmu.2018.01011 PMC5976745

[50] L. A. Mulcahy , R. C. Pink , D. R. Carter , J. Extracell. Vesicles 2014, 3, 24641. 10.3402/jev.v3.24641 PMC4122821 25143819

[51] A. L. Routzahn , P. K. Jain , Nano Lett. 2015, 15, 2504. 25730168 10.1021/acs.nanolett.5b00068

[52] T. Tian , Y. L. Zhu , F. H. Hu , Y. Y. Wang , N. P. Huang , Z. D. Xiao , J. Cell. Physiol. 2013, 228, 1487. 23254476 10.1002/jcp.24304

[53] P. D. Robbins , A. E. Morelli , Nat. Rev. Immunol. 2014, 14, 195. 24566916 10.1038/nri3622 PMC4350779

[54] Y. Wang , L. Wang , C. Chen , X. Chu , Mol. Cancer 2018, 17, 22. 29415727 10.1186/s12943-018-0766-4 PMC5804051

[55] R. U. Takahashi , M. Prieto‐Vila , A. Hironaka , T. Ochiya , Clin. Chem. Lab. Med. 2017, 55, 648. 28231055 10.1515/cclm-2016-0708

[56] S. Wang , A. B. Aurora , B. A. Johnson , X. Qi , J. McAnally , J. A. Hill , J. A. Richardson , R. Bassel‐Duby , E. N. Olson , Dev. Cell 2008, 15, 261. 18694565 10.1016/j.devcel.2008.07.002 PMC2685763

[57] J. E. Fish , M. M. Santoro , S. U. Morton , S. Yu , R. F. Yeh , J. D. Wythe , K. N. Ivey , B. G. Bruneau , D. Y. Stainier , D. Srivastava , Dev. Cell 2008, 15, 272. 18694566 10.1016/j.devcel.2008.07.008 PMC2604134

[58] M. F. Santos , G. Rappa , J. Karbanova , T. Kurth , D. Corbeil , A. Lorico , J. Biol. Chem. 2018, 293, 13834. 30018135 10.1074/jbc.RA118.003725 PMC6130944

[59] G. Rappa , M. F. Santos , T. M. Green , J. Karbanová , J. Hassler , Y. Bai , S. H. Barsky , D. Corbeil , A. Lorico , OncoTargets Ther. 2017, 8, 14443. 10.18632/oncotarget.14804 PMC5362417 28129640

[60] N. C. Meisner‐Kober , M. J. Wood , S. E. L. Andaloussi , D. V. Morrissey , J. Voshol , N. Pizzato , K. Martin , C. Genoud , A. Graff‐Meyer , S. von Bueren , E. Steib , D. Trojer , J. Hean , W. Heusermann , J. Cell Biol. 2016, 213, 173. 27114500 10.1083/jcb.201506084 PMC5084269

