# Supplementary material for: Real‐Time Dissection of the Transportation and miRNA‐Release Dynamics of Small Extracellular Vesicles
Source: Adv Sci (Weinh). 2023 Jan 4;10(7):2205566. doi: 10.1002/advs.202205566 (PMC9982592; doi:10.1002/advs.202205566)
Supplement: Supplementary file 1 — Supporting Information [file ADVS-10-2205566-s004.pdf]

ADVANCED SCIENCE
Open Access

Supporting Information

for *Adv. Sci.*, DOI 10.1002/advs.202205566

Real-Time Dissection of the Transportation and miRNA-Release Dynamics of Small Extracellular Vesicles

Hou-Fu Xia, Zi-Li Yu, Li-Juan Zhang, Shu-Lin Liu, Yi Zhao, Jue Huang, Dan-Dan Fu, Qi-Hui Xie, Hai-Ming Liu, Zhi-Ling Zhang, Yi-Fang Zhao, Min Wu, Wei Zhang, Dai-Wen Pang* and Gang Chen**

Supporting Information

Real-time Dissection of the Transportation and miRNA-release Dynamics of Small Extracellular Vesicles

Hou-Fu Xia, Zi-Li Yu, Li-Juan Zhang, Shu-Lin Liu, Yi Zhao, Jue Huang, Dan-Dan Fu, Qi-Hui Xie, Hai-Ming Liu, Zhi-Ling Zhang, Yi-Fang Zhao, Min Wu, Wei Zhang, Dai-Wen Pang*, Gang Chen**

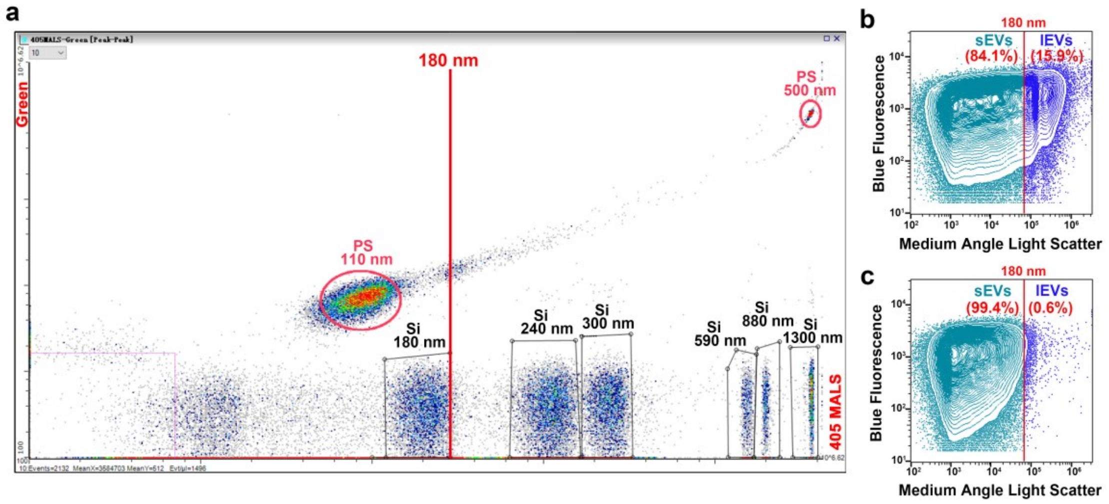

Figure S1. The identification of different sized EVs by Apogee Micro Flow Cytometer. a, Representative color plot showing the general gating strategy used to identify EVs of different size distribution. **b**, The percentage of sEVs and lEVs in pellets obtained from the cell debris-free supernatant by ultracentrifugation at 120,000 g. **c**, The percentage of sEVs and lEVs in pellets obtained from the cell debris-free supernatant by differential centrifugation (16,500 g followed by 120,000 g).

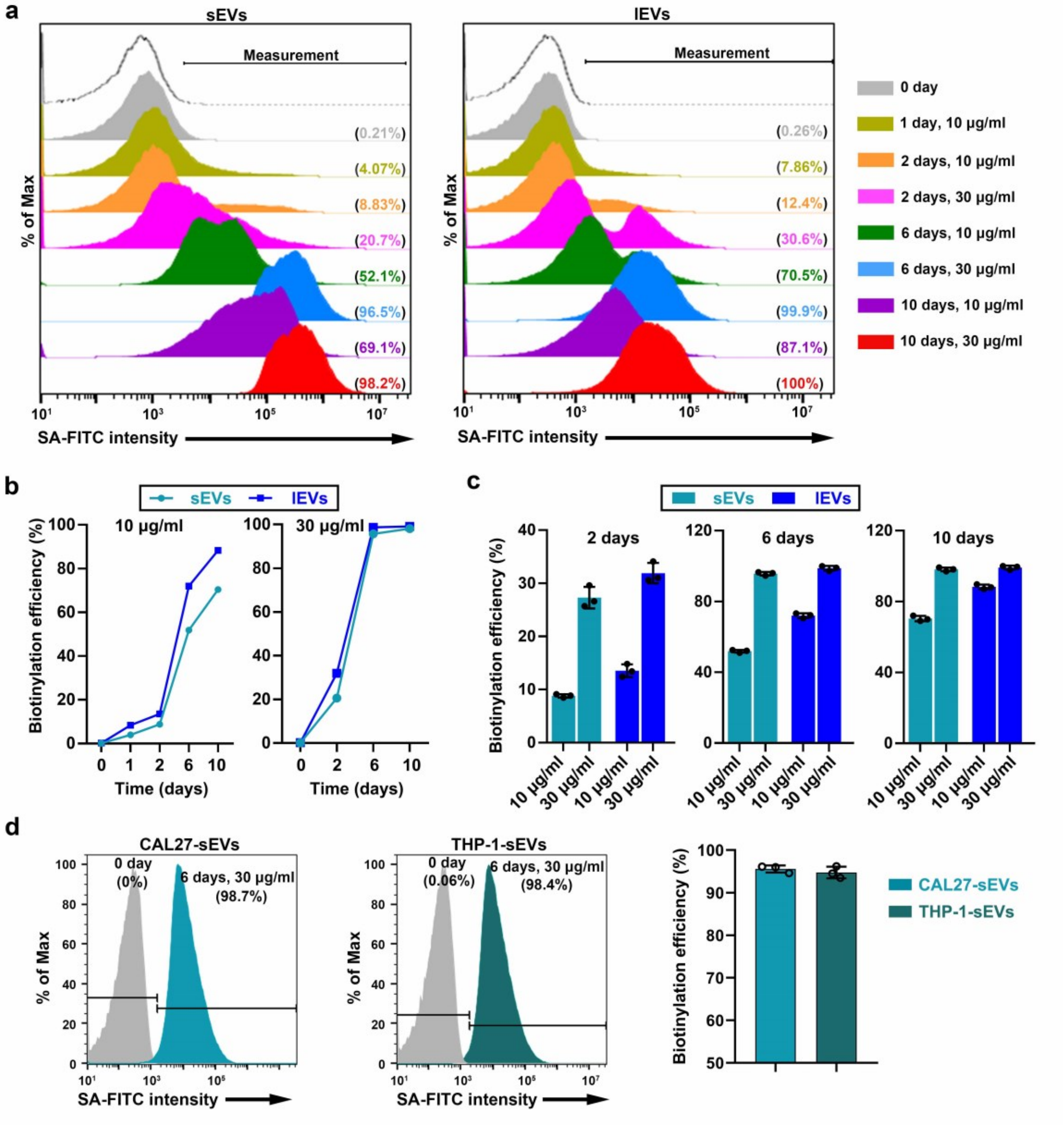

Figure S2. Membrane biotinylation of different EV subtypes. **a**, Biotinylation efficiencies of sEVs and lEVs isolated from the same cell supernatant collected under the indicated conditions. **b**, **c**, Comparison analysis of the biotinylation efficiencies of sEVs and lEVs under indicated conditions (*n* = 3). **d**, Comparison analysis of the biotinylation efficiencies of sEVs of different cellular origins (*n* = 3).

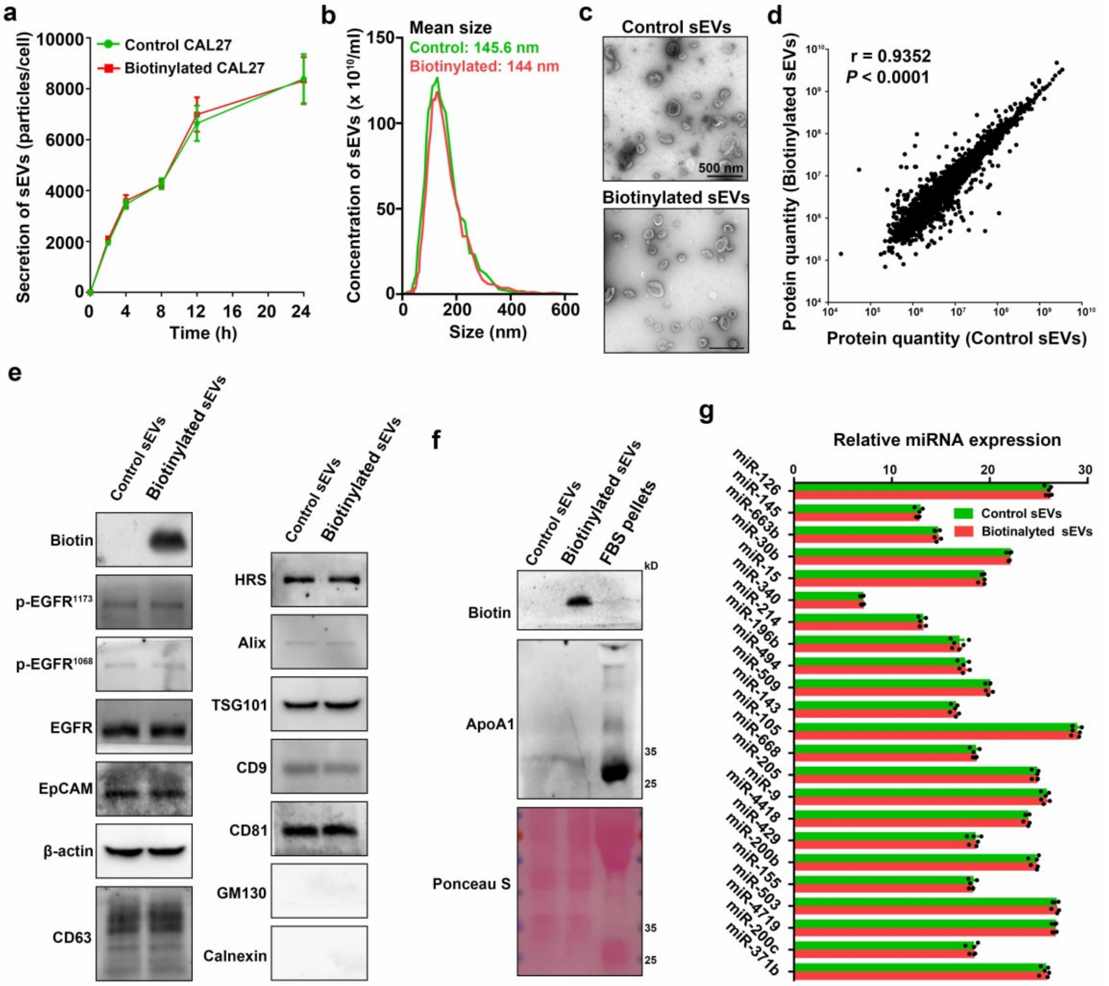

Figure S3. Biofriendly membrane biotinylation of sEVs. **a**, The comparative analysis of sEV secretion level over time of CAL27 cells after culture with (Biotinylated-CAL27) or without (Control CAL27) DSPE-PEG-Biotin (30 μg/ml) for 6 days (n = 3), data are presented as mean ± SD. **b**, The comparative analysis of the size distribution of sEVs isolated from the supernatant of CAL27 cells cultured with (Biotinylated sEVs) or without (Control sEVs) DSPE-PEG-Biotin (30 μg/ml) for 6 days by NTA. **c**, TEM images of the sEVs with (Biotinylated sEVs) or without (Control sEVs) biotinylation. **d**, The total protein expression profile of sEVs with (Biotinylated sEVs) or without (Control sEVs) biotinylation were analyzed by proteomics. **e**, Analyzing the effects of biotinylation on the level of characteristic proteins in sEVs by western blotting, biotinylated sEVs were precipitated with streptavidin affinity gel beads, the same total sEV protein were loaded for each group. **f**, Analyzing the effects of biotinylation on lipoprotein contamination in T-sEVs by western blotting, biotinylated sEVs were precipitated with streptavidin affinity gel beads, the same total sEV protein were loaded for each group. **g**, RT-qPCR analysis of the levels of representative microRNAs in sEVs with (Biotinylated sEVs) or without (Control sEVs) biotinylation (n = 3), data are presented as mean ± SD.

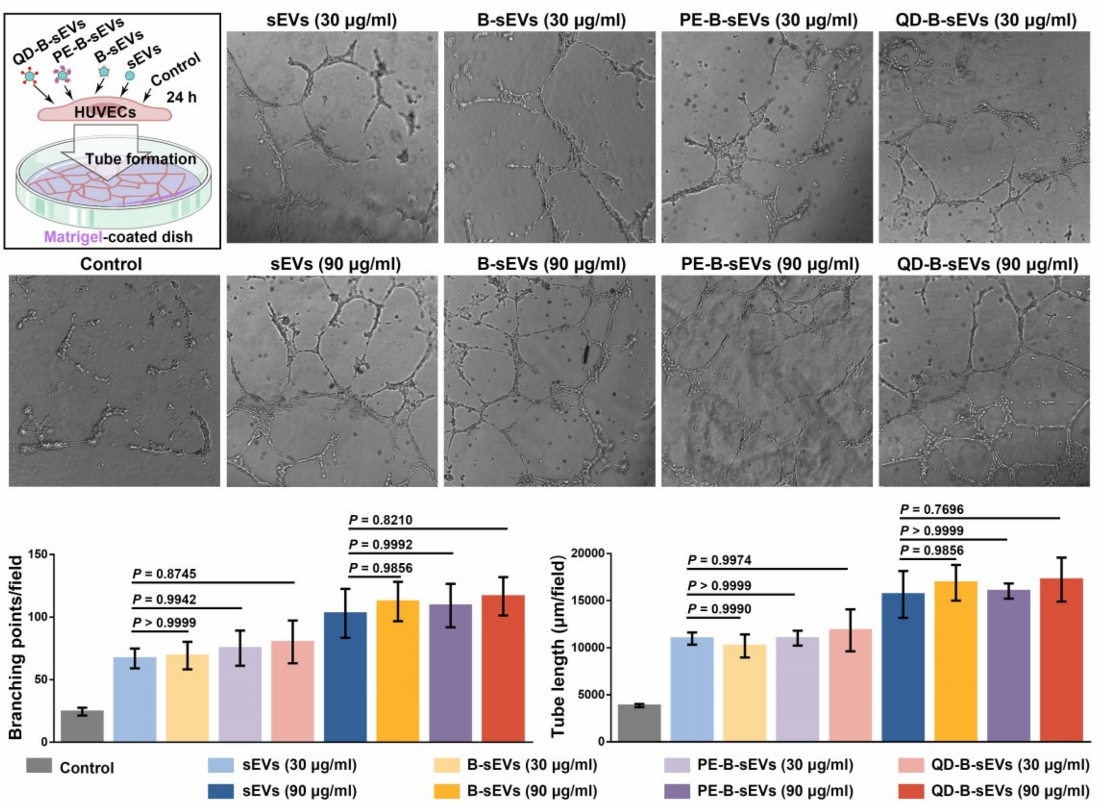

Figure S4. None significant effects of the QD-based labeling strategy on the pro-angiogenic activity of T-sEVs. After co-cultured with 30 μg/ml or 90 μg/ml T-sEVs which were pre-labeled with different tags, ECs were subjected to *in vitro* tube formation assay to compared their angiogenesis capacity. sEVs (control T-sEVs), B-sEVs (biotinylated T-sEVs), PE-B-sEVs (SA-PE-labeled biotinylated T-sEVs), QD-B-sEVs (SA-QD-labeled biotinylated T-sEVs), n = 4, data are presented as mean ± SD, significance was determined using one-way analysis of variance (ANOVA) followed by Tukey's multiple comparison test.

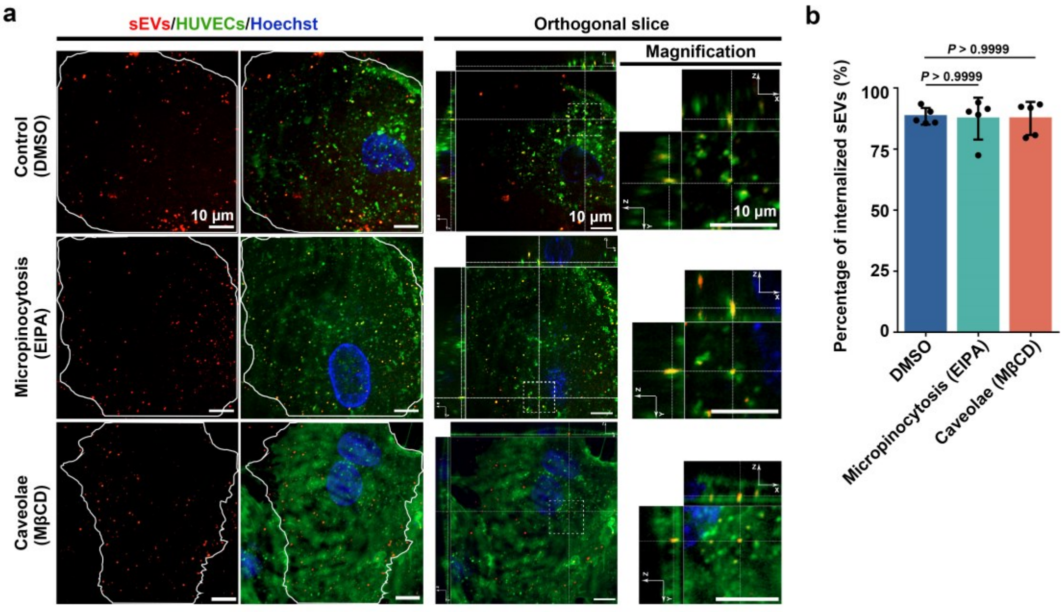

Figure S5. The endocytosis of T-sEVs by ECs. **a**, Representative confocal fluorescence images showing the effects of inhibitors specific to different pathways on the internalization of T-sEVs by ECs. Control, DMSO (Dimethylsulfoxide); Macropinocytosis, EIPA (5-(N-ethyl-N-isopropyl) amiloride, 75 μM); Caveolae, MβCD (Methyl-β-cyclodextrin, 5 mM). **b**, Quantitative analysis of the endocytosis of T-sEVs by ECs shown in (a), $n = 5$, data are presented as mean ± SD, significance was determined using one-way ANOVA followed by Dunn's multiple comparison test.

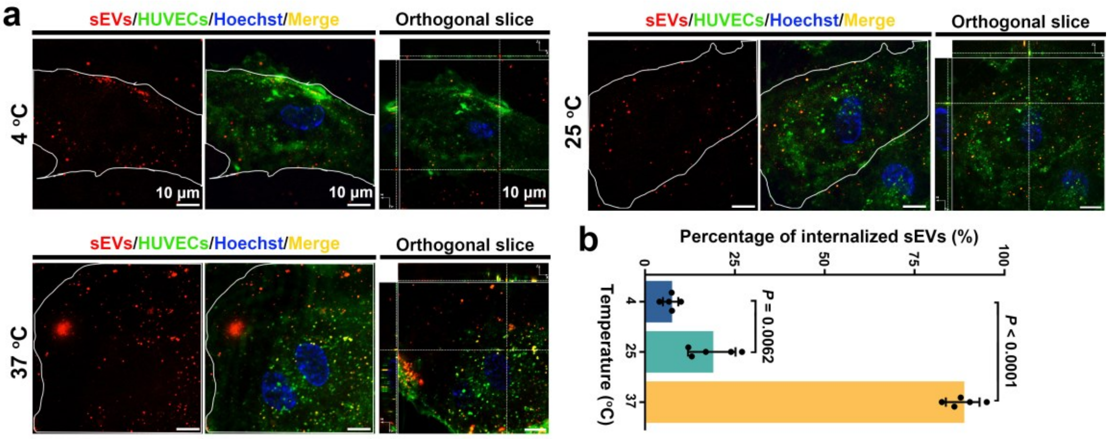

Figure S6. Temperature-dependent endocytosis of T-sEVs by ECs. a, Representative confocal fluorescence images of the endocytosis of T-sEVs by ECs at different incubation temperature (4 °C, 25 °C and 37 °C). **b**, Quantitative analysis of the endocytosis of T-sEVs by ECs shown in (a), $n = 5$, data are presented as mean ± SD, significance was determined using one-way ANOVA followed by Tukey's multiple comparison test.

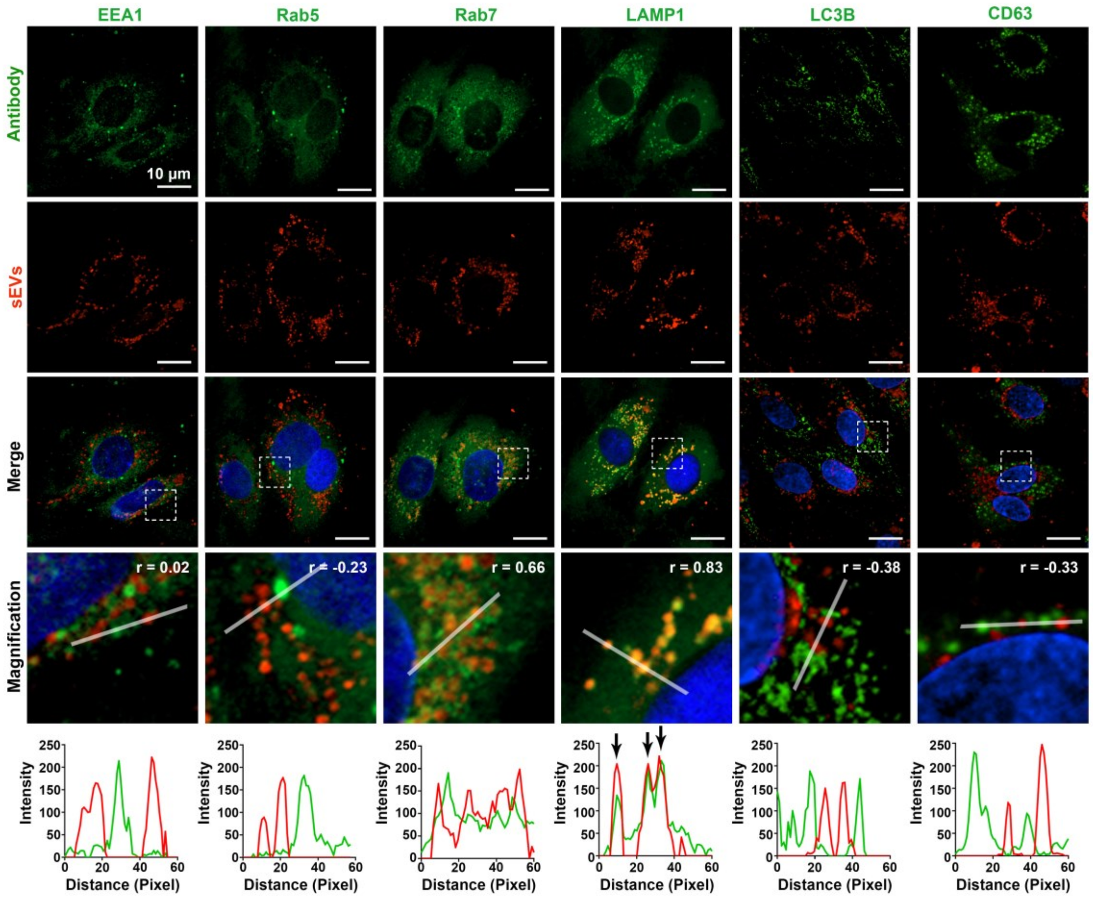

Figure S7. The destination of T-sEVs in ECs. ECs were co-cultured with QD-labeled T-sEVs at 37 °C for 6 h, followed by staining with various markers after fixation. Representative confocal fluorescence images and line profile analyses demonstrated the co-localization of T-sEVs with LAMP-1^+ endosomes in ECs, the Pearson's correlation coefficient (r) was calculated with Image J.

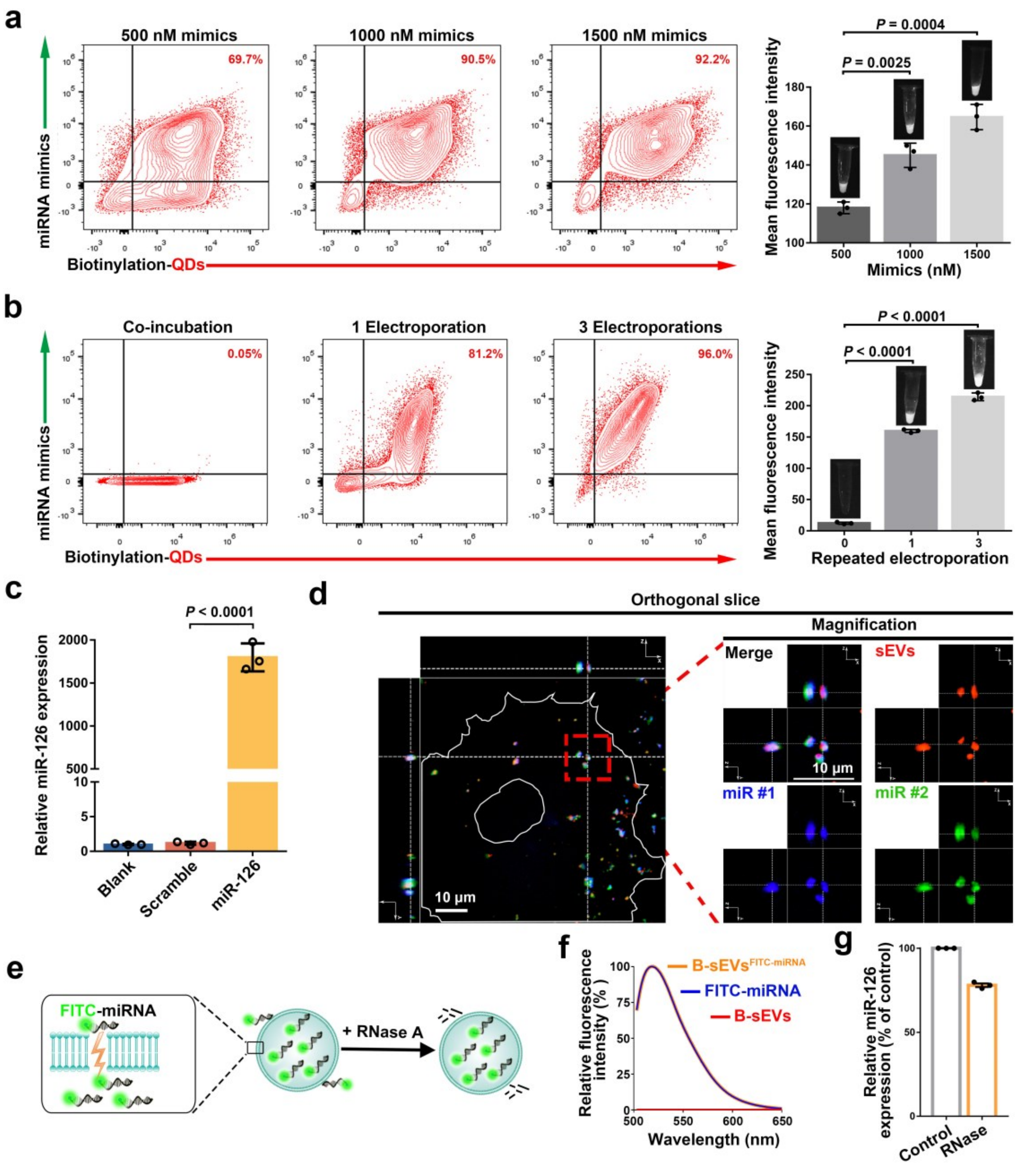

Figure S8. The optimization of the multi-color and multi-component labeling of sEVs. a, Flow cytometric analysis and fluorescence spectrophotometer analysis of miRNA loading efficiency in sEVs at indicated concentrations, $n = 3$, data are presented as mean ± SD, significance was determined using one-way ANOVA followed by Tukey's multiple comparison test. **b**, Flow cytometric analysis and fluorescence spectrophotometer analysis of miRNA loading efficiency in sEVs under different electroporation conditions, $n = 3$, data are presented as mean ± SD, significance was determined using two tailed unpaired *t*-test. **c**, The loading efficiency of miR-126 into T-sEVs by electroporation was analyzed by RT-qPCR. T-sEVs were electroporated with RNase-free water (Blank), 1000 nM scramble miRNA (Scramble) or 1000 nM miR-126 (miR-126) under the same condition, $n = 3$, data are presented as mean ± SD, significance was determined using two tailed unpaired *t*-test. **d**, Orthogonal slice images of multi-labeled sEVs (QD-labeled membrane, AF405-labeled miR #1, FAM-

labeled miR #2) in ECs. **e**, Schematic diagram for the RNase protection assay. **f**, Fluorescence emission spectrum of biotinylated T-sEVs with (B-sEVs$^{\text{FITC-miRNA}}$) or without (B-sEVs) electroporation with FITC-labeled miRNA. **g**, The level of miR-126 in electroporated T-sEVs with (RNase) or without (Control) RNase A (10 μg/ml) incubation were analyzed by RT-qPCR, *n* = 3, data are presented as mean ± SD.

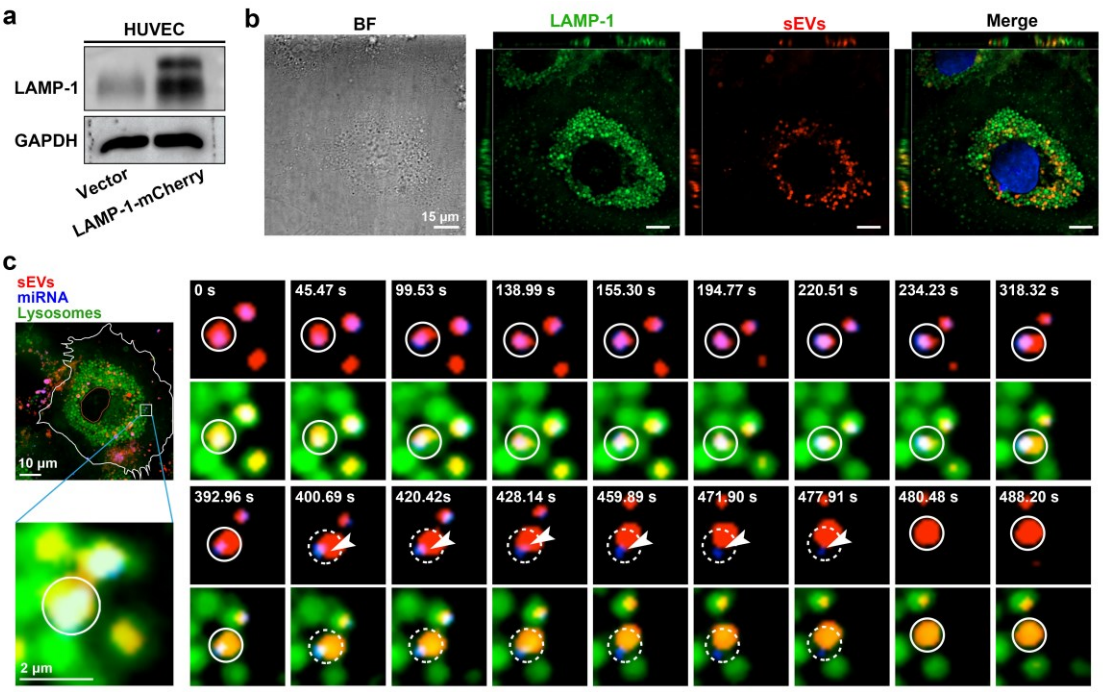

Figure S9. The miRNA release behaviors of T-sEVs in ECs. a, ECs were labeled with fluorescent protein-tagged LAMP-1 (LAMP-1-mCherry). **b**, Representative confocal fluorescence images showing the location of T-sEVs (red) in LAMP-1^{+} lysosomes (green pseudo-color) of ECs after 6 h. **c**, Snapshots of the miRNA-release process of a dual-color labeled T-sEV (QDs-labeled membrane and AF405-labeled miR-126) shown in Video S7, white arrows indicated the transient retention of miRNA (blue) after the signal separation.

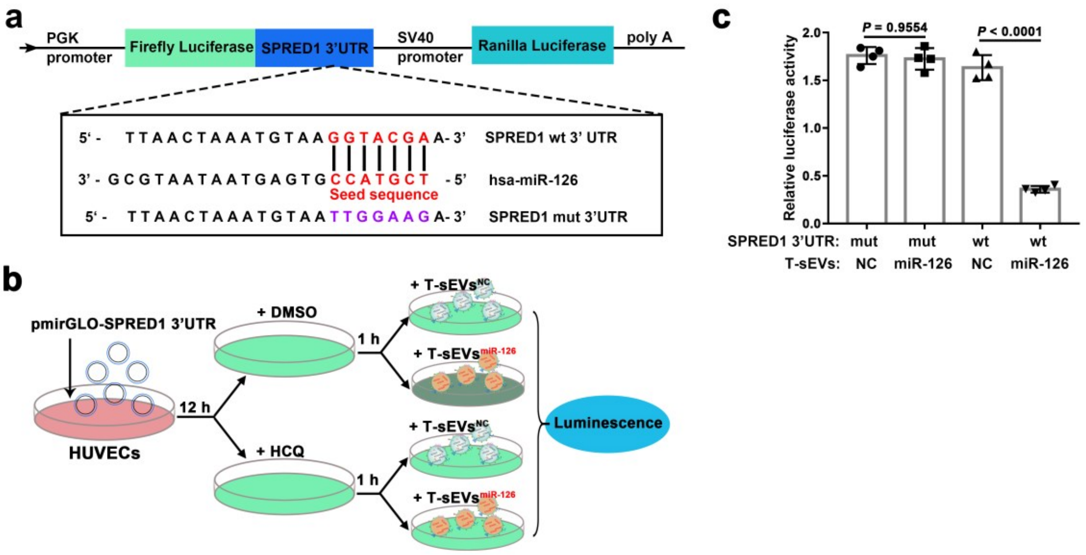

Figure S10. T-sEVs delivered functional miRNA cargos to recipient ECs. a, Strategy to generate the miR-126-targeted Dual-Luciferase reporter assay system. Based on the pmirGLO vector, the normal SPRED1 3'UTR (SPRED1 wt 3'UTR) or the mutant SPRED1 3'UTR (SPRED1 mut 3'UTR, from GGTACGA to TTGGAAG) were separately fused to the firefly luciferase reporter. **b**, The flow diagram about the function assay of sEVs-delivered miR-126. The ECs were firstly transfected with pmirGLO-SPRED1 wt 3'UTR or pmirGLO-SPRED1 mut 3'UTR, then pre-treated with DMSO or HCQ (100 μM) for 1 h and followed by co-incubation with negative control mimics-loaded T-sEVs (T-sEVs^{NC}) or miR-126 mimics-loaded T-sEVs (T-sEVs$^{miR\text{-}126}$). All the groups were finally lysed and subjected to the luminescence scan after 12 h. **c**, miR-126 mimics-loaded T-sEVs (T-sEVs$^{miR\text{-}126}$) significantly repressed the expression level of SPRED1 wt 3'UTR luciferase, rather than SPRED1 mut 3'UTR luciferase, n = 4, data are presented as mean ± SD, significance was determined using one-way ANOVA followed by Tukey's multiple comparison test.

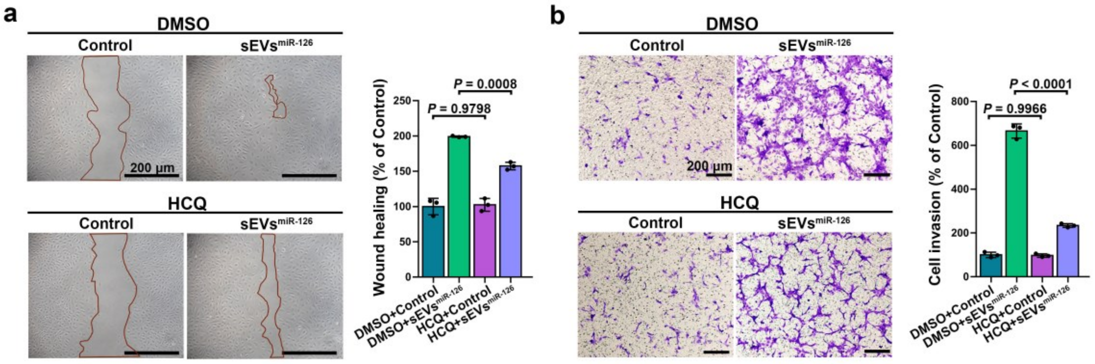

Figure S11. Disrupting the lysosome acidification impaired the T-sEVs-mediated pro-angiogenic effects on ECs. ECs were pretreated with 100 μM Hydroxychloroquine Sulfate (HCQ) or DMSO (DMSO) for 1 h and co-cultured with T-sEVs, which were pre-loaded with miR-126 (sEVs$^{miR\text{-}126}$), then the angiogenic capacity of ECs was evaluated by wound healing assay (**a**) and boyden chamber assay (**b**), n = 3, data are presented as mean ± SD, significance was determined using one-way ANOVA followed by Tukey's multiple comparison test.

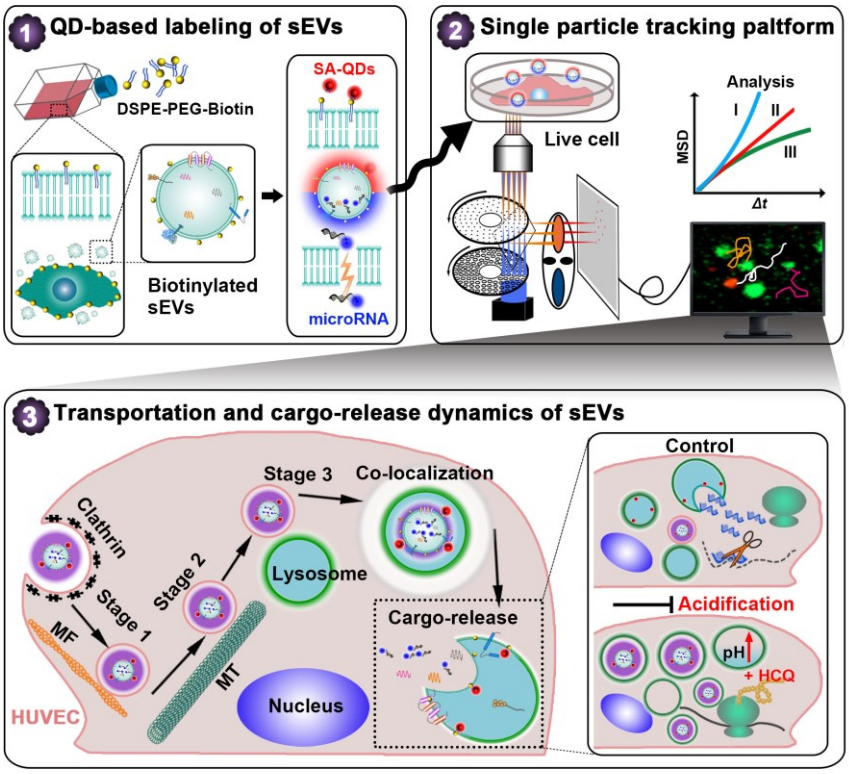

Figure S12. Schematic of quantum dot-based single particle tracking for dissecting the transportation and cargo-release dynamics of small extracellular vesicles.

Video S1. The coincident movement of QD-labeled EVs (red) and CellMask-positive endocytic vesicles (green) in EC.

Video S2. Tracking the clathrin-dependent entry of QD-labeled EVs (red) in ECs expressing Clathrin light chain β-EGFP (green).

Video S3. The QD-labeled T-sEV (red) moved directionally in the cytoplasm and finally reached the perinuclear region in EC.

Video S4. The QD-labeled T-sEV (red) moved directionally in the cytoplasm and finally reached the perinuclear region in EC.

Video S5. The typical "slow-fast-slow" transportation of a QD-labeled T-sEV (red) in EC.

Video S6. Tracking the co-localization process of the QD-labeled T-sEV (red) and an acidic endosome (green) in EC.

Video S7. Tracking the miRNA (blue) release behavior of the QD-labeled T-sEV (red) in acidic endosome (green) of EC.

Video S8. Tracking the miRNA (blue) release behavior of the QD-labeled T-sEV (red) in a LAMP-1^+ lysosome (green pseudo-color) of EC.

Video S9. Tracking the stable co-localization of miRNA (blue) and QD signal (red) in lysosome (green pseudo-color) of HCQ-treated ECs.
